# Retinoic acid-inducible gene-I aggravates neuroinflammation in early brain injury after subarachnoid hemorrhage through mediating brain microvascular endothelial cell pyroptosis

**DOI:** 10.1016/j.neurot.2025.e00572

**Published:** 2025-04-02

**Authors:** Bowen Sun, Yuchen Li, Shuai Lan, Xi-ao Wang, Yeping Ling, Harshal Sawant, Bohan Zhang, Jinshuo Yang, Jinju Wang, Pei Wu, Shancai Xu, Ji Bihl, Huaizhang Shi

**Affiliations:** a Department of Neurosurgery, The First Affiliated Hospital, Harbin Medical University, Harbin 150001, Heilongjiang, China; b Departments of Biomedical Sciences, Joan C. Edwards School of Medicine, Marshall University, Huntington, WV 25755, USA; c Department of Physiology and Pharmacology, Loma Linda University, Loma Linda, CA 92354, USA

**Keywords:** Subarachnoid hemorrhage, Neuroinflammation, Brain microvascular endothelial cells, Pyroptosis, Retinoic acid-inducible gene I

## Abstract

Retinoic acid-inducible gene-I (RIG-I) is an immune signal that promotes inflammatory responses and plays an important role in endothelial cell-mediated inflammation. Currently, no studies have investigated the role of RIG-I in early brain injury (EBI) after subarachnoid hemorrhage (SAH). In this research, an *in vivo* SAH model was established in Sprague Dawley (SD) rats through carotid artery puncture, while oxyhemoglobin (OxyHb) was used to stimulate brain microvascular endothelial cells (BMVECs) to generate an *in vitro* SAH model. The results showed that RIG-I was activated and expressed in BMVECs in both *in vivo* and *in vitro*. To explore how RIG-I is involved in the EBI, small interfering RNA was used to downregulate its expression. Compared with SAH rats, RIG-I knockdown rats had better short-term and long-term neurological recovery after SAH, milder brain edema and neurodegeneration, and weaker blood-brain barrier disruption and neuroinflammation. Furthermore, RIG-I knockdown attenuated SAH-induced BMVECs pyroptosis. In OxyHb-stimulated BMVECs, RIG-I knockdown reduced the cellular dysfunction and inflammation. To determine the mechanism of RIG-I in BMVECs pyroptosis, co-immunoprecipitation was used to verify the direct binding of RIG-I to caspase-1, and RIG-I knockdown could reduce Oxy-Hb-induced BMVECs pyroptosis, while caspase-1 inhibitor VX-765 can alleviate the tight junction loss, inflammation and pyroptosis exacerbated by RIG-I agonist. In addition, RIG-I levels in the cerebrospinal fluid of SAH patients were higher than those in controls and correlated with the levels of pyroptosis, inflammatory factors and clinical outcomes. In summary, the results demonstrated that RIG-I aggravates neuroinflammation after SAH by promoting caspase-1-mediated BMVECs pyroptosis.

## Introduction

Although subarachnoid hemorrhage (SAH) ranks third in terms of incidence among different stroke subtypes, its alarming mortality and disability rates have led many to consider it the most dangerous cerebrovascular disease [[1], [2], [3]]. The rupture of intracranial aneurysms causing blood to intrude into the subarachnoid space is the main cause of SAH [4]. Timely aneurysm treatment decreases the risk of rebleeding after SAH and effectively reduces mortality, but the blood remaining in the subarachnoid space still causes serious damage to the central nervous system [5,6]. Recent studies have suggested that early brain injury (EBI) occurring within 72 ​h after SAH is an important factor causing poor patient outcomes, including microcirculatory dysfunction, blood-brain barrier (BBB) disruption, neuroinflammation, brain edema, oxidative cascade, and brain cell death [5].

Traditionally, brain microvascular endothelial cells (BMVECs) have been a major component of the BBB, a critical barrier that effectively limits the entry of potentially neurotoxic plasma components, blood cells, and pathogens into the brain [7,8]. It can also sense signals, for instance, the breakdown products of blood in CSF following SAH serve as a representative example.danger-associated molecular patterns (DAMPs) and pathogen-associated molecular patterns(PAMPs), and release various pro-inflammatory factors that participate in the inflammatory response process [[9], [10], [11]]. Pyroptosis is a programmed cell death mode with pro-inflammatory effects, which has been shown to play an important role in the development of SAH, but existing studies have predominantly focused on the role of microglia and neuronal pyroptosis after SAH [12,13]. Thus, current studies on BMVECs pyroptosis after SAH are very limited. Hu et al. first reported that huperzine A can improve BBB damage, neuronal apoptosis, and neurological deficits after SAH by inhibiting BMVECs pyroptosis, but the upstream mechanism of BMVECs pyroptosis after SAH needs to be further explored [14].

Retinoic acid-inducible gene I (RIG-I) is an important immune gene that usually functions as a cytosolic pattern recognition receptor that can be activated by viral RNA and perform immune surveillance to promoteantiviral response [15]. In nonviral diseases, RIG-I can act as a DAMP sensor to promote inflammatory responses, such as being activated by myoglobin to promote NF-κB/caspase-3 signaling in the acute kidney injury model [16]. In central nervous system diseases, RIG-I is expressed in pathological brain sections of Alzheimer's disease patients and is also involved in the innate immune response after cerebral ischemia [17,18]. However, there have been no studies investigating the role of RIG-I in SAH. In recent years, RIG-I has been considered important for controlling disorders associated with altered immunity and inflammation in peripheral endothelial cells (ECs). RIG-I activation can significantly impair ECs function and induce the activation of downstream pro-inflammatory signals, further participating in vascular pathology or barrier function damage [10]. The present study hypothesized that RIG-I in BMVECs could be activated by DAMPs, such as blood breakdown products after SAH, and promote EBI.

Caspase-1 activation can promote the maturation of interleukin (IL)-1β and IL-18 and cleave gasdermin D (GSDMD), further encouraging cell pyroptosis [19]. It is currently known that caspase-1 inflammasome activation leads to post-SAH neuroinflammation, and caspase-1 inhibition can potently reduce endothelial inflammation in BBB damage induced by the organophosphate paraoxon [20,21]. However, the role of caspase-1 in BMVECs pyroptosis after SAH is unknown. More importantly, existing research shows that caspase-1 is activated after RIG-I recognizes RNA viruses and there is a direct relationship between the two [22]. In addition, myoglobin can promote the interaction between RIG-I and caspase-1 in macrophages, thereby promoting cell polarization to the M1 type and pyroptosis [23]. The role of the interaction between RIG-I and caspase-1 in BMVECs pyroptosis and brain injury after SAH is unknown.

Therefore, the present study examined the association between RIG-I and pathways related to BBB damage and pyroptosis to explore the role of RIG-I in SAH. It was suggested that RIG-I activation mediated the maturation of caspase-1 inflammasome and GSDMD cleavage in BMVECs, leading to BBB leakage and BMVECs pyroptosis causing neuroinflammation after SAH.

## Material and methods

### SAH patients and cerebrospinal fluid sampling

The present investigation received approval and registration from the Ethics Committee of the First Affiliated Hospital of Harbin Medical University (2021136). The study comprised 28 patients with SAH admitted to the Neurosurgery Department of the First Affiliated Hospital of Harbin Medical University between December 2022 and December 2023. Informed consent was obtained from the patients or their families. Cerebrospinal fluid (CSF) was collected via lumbar or ventricular puncture within 72 ​h of SAH onset depending on the patient's state and subsequently centrifuged at 10,000×*g* for a minimum of 10 ​min at 4 ​°C, and stored at −80 ​°C. Furthermore, CSF from 12 patients with normal pressure hydrocephalus served as the control group. The modified Rankin scale (mRS) was employed to assess patient outcomes at the 6-month follow-up, and a favorable outcome was classified as mRS of 0–2 (Table S1) [24].

### Animal models of SAH

The SAH model was created using internal carotid artery puncture in 8-week-old male Sprague-Dawley (SD) rats (250–300 ​g) obtained from the Animal Center of the First Affiliated Hospital of Harbin Medical University. All the rats were in healthy status with normal functioning immune system. The rats had received no genetic modifications or any procedures previously. All the *in vivo* experiments complied to the ARRIVE guidelines [25]. The particular modeling technique employed in the study was based on prior research [26,27]. Rats were assigned codes and randomized before surgery, followed by anesthesia via intraperitoneal injection of phenobarbital (50 ​mg/kg). A sharp 4-0 monofilament nylon suture was employed for insertion into the left internal carotid artery at the carotid bifurcation. The nylon thread was advanced until reaching the junction of the anterior cerebral and middle cerebral arteries, when resistance was met. At that moment, the vessel was pierced and the nylon thread was extracted. The sham operation group employed the identical technique as previously described, but refrained from puncturing the blood vessels. The skin incision was sutured post-procedure, and the rats were positioned on a heating pad at 37.5 ​°C, with their vital signs being continually monitored. Postoperatively, all animals have unrestricted access to food and water and are housed individually in appropriate conditions of temperature and humidity within their enclosures.

### Intracerebroventricular injection

RIG-I small interfering RNA (siRNA, Haixing Biosciences, Shanghai, China) was employed to attenuate RIG-I gene expression *in vivo*. The intracerebroventricular (i.c.v.) siRNA method was conducted as previously outlined [27]. Rats were administered pentobarbital (40 ​mg/kg) via intraperitoneal injection and positioned in a stereotaxic device. A 10-μL microsyringe was subsequently introduced into the left ventricle at the following precise coordinates relative to the bregma: 1.0 ​mm laterally, 1.5 ​mm posteriorly, and 3.1 ​mm beneath the dural surface. During a 48-h window before surgery, 500 ​pmol siRNA was administered i.c.v. at a rate of 0.3 ​μL/min, for a total volume of 5 ​μL. An equivalent volume of scramble siRNA was utilized as a negative control (NC). RNase-Free water was used as the carrier of the siRNA [28].

In addition, to investigate the mechanism of RIG-I and Caspase-1 involvement in EBI, RIG-I activator 5′ppp-dsRNA/LyoVec™ and Caspase-1 inhibitor VX-765 were also injected into the ventricles in the aforementioned manner. Among them, 5′pp dsRNA was carried by sterile PBS, while VX-765 was carried by corn oil.

### Experimental design

All rats in the investigation were randomly allocated and detailed experimental design is outlined in the supplementary file (Fig. S1). Rats were randomized by using the simple randomization.

### SAH grading

The SAH grade was evaluated by two separate researchers who were unaware of the animals’ classifications. The evaluation criteria aligned with those of prior studies (Fig. S2) [29]. The basal region of the rat brain was categorized into six areas, each evaluated on a scale from 0 to 3 based on the extent of subarachnoid hemorrhage, resulting in a cumulative score for the six regions ranging from 0 to 18. Consequently, rats with scores <8 were excluded from the study. The dead rats were excluded from the study and were recorded separately.

### Behavioral tests

Neurological deficits were assessed daily using the Garcia score and beam balance test as previously outlined (Tables S2 and S3) [30,31]. The Garcia score has six assessments that examine motor and sensory skills, including spontaneous activity, limb movement symmetry, forepaw extension, climbing ability, body proprioception, and responsiveness to vibrissae stimulation. The rotarod test utilized Rotamex-5 equipment (Columbus Instruments, Columbus, OH, USA) to evaluate physical strength, balance, and coordination. The mice underwent pre-training for three consecutive days, and baseline performance was assessed one day before surgery. Acclimatization involved three trials during which the speed was incrementally elevated from 4 to 40 ​rpm over a duration of 5 ​min.

### Morris water maze

The Morris water maze served as an assessment tool for long-term neurological function in rats, specifically evaluating spatial learning and memory, as detailed in prior research [32]. The experiment was conducted between days 22 and 28 following SAH induction by researchers blinded to the experimental group assignments. Following the visible platform test on day 22, rats were instructed to locate a circular platform with a diameter of 10 ​cm and positioned 2 ​cm beneath the liquid surface in a circular pool measuring 180 ​cm in diameter. The platform remained concealed for the subsequent five days, and the experiment was executed thrice daily. Animals were instructed to remain on the platform for 10 ​s after locating it or being directed to it. On day 28, the platform was dismantled, permitting the animals to swim from a designated starting point for 120 ​s. Multiple parameters were documented for statistical analysis, including escape delay, platform crossings, swimming distance, velocity, and trajectory.

### Brain water content

After removing the clotted blood, the brain was initially weighed in its moist state to ascertain the wet weight. Following a 12-h incubation in the oven at 100 ​°C, the tissue was reweighed to determine dry weight, and brain water content percentage was estimated using the formula [(wet weight - dry weight)/wet weight] ​× ​100 ​%.

### Hematoxylin and eosin staining

After dewaxing and hydration, paraffin sections were subjected to staining with hematoxylin and eosin (H&E) and subsequently mounted with neutral gum following dehydration achieved using the following escalating ethanol concentrations: 75 ​% alcohol (5 ​min), 85 ​% alcohol (5 ​min), eosin (5 ​min), 95 ​% alcohol (1 ​min, repeated twice), 100 ​% alcohol (10 ​min, repeated twice), and xylene (10 ​min, repeated twice). Tissue slices were examined with a light microscope (Leica, Wetzlar, Germany). Regions of interest selected for image acquisition are described in Fig. S3.

### Evans blue extravasation test

The BBB permeability was assessed using Evans blue (Sigma-Aldrich, St. Louis, MO, USA) dye extravasation 24 ​h after SAH. Following anesthesia with chloral hydrate, rats in each cohort were subsequently administered 2 ​% evans blue via intravenous injection. After 60 ​min, the rat brain was weighed following transcardial perfusion with saline and homogenized in 50 ​% trichloroacetic acid. The brain tissue was then homogenized and the supernatant was collected post-centrifugation, combined with ethanol and TCA, and incubated overnight at 4 ​°C. The Evans blue concentration in the supernatant was ascertained by measuring the absorbance at 630 ​nm with a microplate reader (BioTek, Santa Clara, CA, USA).

### Cell culture and SAH model in vitro

Primary human BMVECs (hBMVECs, ACBRI 376; Cell System, Kirkland, WA, USA) were cultured in complete medium until reaching confluence according to the methodology outlined by Cell Systems and maintained in complete medium with 5 ​% CO2 at 37 ​°C. The complete culture medium comprised 10 ​% serum and was supplemented with 5 ​mL of CultureBoost™ and 10 ​mL of Attachment Factor™ for each 500 ​mL of the cell system culture media (Cell Systems, USA). Cells were exposed to serum-free media supplemented with 10 ​μM oxyhemoglobin (Sigma-Aldrich, USA) for 24 ​h to simulate the SAH model *in vitro* as described [33]. SiRNA (Santa Cruz Biotechnology, Santa Cruz, CA, USA) was administered to the culture medium before the introduction of OxyHb and following the manufacturer's instructions to suppress RIG-I expression. Briefly, the hBMVECs were transfected with 30 ​pmol control siRNA or RIG-I siRNA using siRNA transfection reagent in siRNA transfection medium for 6 ​h and complete medium for 48 ​h [34,35]. The knockdown effect was confirmed based on the Western blot data. To activate RIG-I, its agonist, 1 ​μg/mL of 5′ ppp-dsRNA/LyoVec™, and Control were added to the culture medium 30 ​min after the addition of OxyHb [36]. In addition, to inhibit caspase-1 in the mechanism study, its inhibitor VX-765 (10 ​μM; InvivoGen, San Deigo, CA, USA) was dissolved in dimethyl sulfoxide (DMSO) and added 2 ​h before adding OxyHb [37].

### Cell viability

The cell viability of each group was assessed using the 3-(4,5-Dimethylthiazol-2-yl)-2,5-Diphenyltetrazolium Bromide (MTT, Sigma-Aldrich, USA) assay as outlined in a prior report [38]. Cells were inoculated onto 96-well plates with 100 ​μL of full culture medium at a concentration of 2 ​× ​10^3^ ​cells per well. Following co-incubation with the designated treatments, MTT solution (20 ​μL, 5 ​mg/mL) was introduced to each well and incubated at 37 ​°C for 4 ​h. Subsequently, 150 ​μL of DMSO was added to each well and incubated at 37 ​°C for 30 ​min. Measurements were obtained using a microplate reader. Optical density (OD) was measured at 490 ​nm utilizing duplicate wells for each set.

### Cell cytotoxicity assay

The assessment of cellular cytotoxicity was conducted using a lactate dehydrogenase (LDH) kit (Fisher Scientific, Waltham, MA, USA). Cells were grown in 96-well plates, with three replicate wells per group. Following the designated treatments, the cells were utilized for the LDH activity assay. The optical density was assessed at 490 ​nm and 680 ​nm using a microplate reader, and the calculations for percent cytotoxicity were performed according to the manufacturer's guidelines.

### Wound healing test

Wound healing assays were conducted to investigate the impact of various treatments on BMVECs migratory function. BMVECs were inoculated into 6-well plates and incubated for 24 ​h. Subsequently, an incision was made using a 1-mL sterile pipette tip. Cells were categorized and processed as previously described, and wound images were captured using a microscope 0, 12, and 24 ​h post-incubation, with each grouping replicated six times. ImageJ software (NIH, Bethesda, MD, USA) was employed to assess and quantify the extent of wound healing.

### Tube formation

The tube formation experiment was carried out to assess the cellular function of hBMVECs as previously described [39]. Matrigel solution (Corning, Glendale, AZ, USA) was applied to a 24-well plate on ice (250 ​μL per well) and incubated at 37 ​°C for 30 ​min to achieve solid gel formation. Following the established protocol for growing hBMVECs, 300 ​μL of culture medium containing ∼4 ​× ​10^5^ ​cells/mL of hBMVECs was inoculated into a 24-well plate with a solidified gel and introduced the relevant therapies to the culture medium. Imaging was conducted 12 ​h later and lumen development was statistically evaluated utilizing ImageJ.

### Immunofluorescence staining

Rats from each group received anesthesia and underwent perfusion with cold normal saline, followed by 4 ​% cold paraformaldehyde (PFA) for tissue fixation. Brains were extracted and post-fixed in 4 ​% paraformaldehyde for 24 ​h, dehydrated in 30 ​% sucrose for 72 ​h, and then sectioned into serial 15-μm-thick coronal slices of the basal temporal lobes utilizing a cryomicrotome (Leica). The slices were incubated overnight at 4 ​°C with the specified primary antibodies for immunofluorescence labeling (Table S6). The slices were then incubated with fluorescence-conjugated secondary antibodies for 1 ​h at ambient temperature. Nuclei were subsequently stained with 4′,6-diamidino-2-phenylindole (DAPI) (Solarbio, Beijing, China) mounting solution after three washes with phosphate-buffered saline (PBS) at ambient temperature. Fluorescence microscopy was used to observe and capture images (Leica). The regions of interest selected for image acquisition are shown in Fig. S3. *In vitro* research involved seeding cells on glass coverslips followed by three gentle washes with PBS to remove any leftover culture material. The cells were then fixed in 4 ​% PFA for 20 ​min, permeabilized with 0.3 ​% Triton X-100 for 15 ​min, and washed with 5 ​% donkey serum for blocking for 1 ​h. Next, the cells were incubated overnight at 4 ​°C with specific primary antibodies at a 1:100 dilution. The subsequent procedures corresponded to the *in vivo* experiments.

### Evans blue fluorescence labeling

We learned the method from the articles that have been published and refined it to better align with our needs [40]. Evans blue solution (4 ​% in saline) was injected intravenously (2.5 ​g/mL) through the tail vein. After 60 ​min, the rats were transcardially perfused for 2 ​min with 4 ​°C saline followed by 10 ​% formalin neutral buffer solution.

After perfusion, the brains were coronally sectioned into slices of 10 ​μm thickness using a brain slicer according to the method mentioned above. Nuclei were subsequently stained with DAPI. Fluorescence microscopy was used to observe and capture images.

### Fluoro-Jade C

Fluoro-Jade C (FJC) staining was used to detect degenerated neurons and was carried out according to the instructions for the FJC Staining Kit (Millipore, St. Louis, MO, USA). Frozen slides were immersed in 1 ​% sodium hydroxide solution, 70 ​% ethanol, and 0.06 ​% potassium permanganate solution. Subsequently, sections were incubated with 1:10,000 FJC solution. Fluorescence microscopy was utilized to observe and collect images. Select regions of interest were chosen for image acquisition and quantitative analysis. Finally, FJC positive cells were counted and analyzed by a blind observer using Image J software.

### Enzyme linked immunosorbent assay (ELISA)

Cytokines of interest were identified utilizing ELISA kits according to the manufacturer's guidelines (Table S4). IL-1β and TNF-α were identified in the homogenate of temporal lobe tissue on the ipsilateral side in rats and hBMVECs culture supernatants to investigate the impact of RIG-I on neuroinflammation. Furthermore, levels of RIG-I, caspase-1, and IL-1β in human CSF were assessed to ascertain their associations. Detailed information on various ELISA kits and their corresponding serial numbers is provided in Table S4 [41].

### Western blotting

The total protein retrieved from the damaged side of the brain was synthesized using radio-immunoprecipitation assay (RIPA) lysis buffer, separated using SDS-PAGE, and transferred to a PVDF membranes. The membranes were treated with primary antibodies (Table S4) overnight at 4 ​°C. On the next day, the samples were washed three times for 10 ​min each time. Subsequently, the membranes were incubated with secondary antibodies (Table S4) conjugated to horseradish peroxidase for at least 1 ​h at room temperature. The samples were then washed again using the same method described above. The test technique employed an ECL chemiluminescent substrate to identify the immune response bands. The analysis was conducted using the Image J program.

### Co-immunoprecipitation (Co-IP)

The hBMVECs were lysed in the IP buffer (Thermofisher, Waltham, MA, USA) for 30 ​min and centrifuged at 12,500 ​rpm for 15 ​min after extraction. A/G magnetic beads were preincubated with specific antibodies for 6 ​h and subsequently washed three times. The cell lysate was then added and incubated for 2 ​h. The magnetic beads were rinsed three times. Next, the immunoprecipitates were eluted by boiling with 1 ​% SDS sample buffer for 10 ​min, and the supernatant was collected after centrifugation at 12,500 ​rpm for 2 ​min. Western blot was then utilized to determine the binding affinity between the two proteins.

### Statistical analysis

All results were analyzed using GraphPad Prism software (version 5.04) and SPSS software. The results are presented as mean ​± ​standard deviation. The data will be disposed and excluded if the assumptions were not met. The differences between the two groups were assessed utilizing Student's *t*-test. Group differences were determined using one-way ANOVA and Tukey's post hoc test. The Pearson's correlation test was employed to evaluate the association between RIG-I in human CSF and caspase-1, IL-1β, and mRS scores. A *P* value below 0.05 was considered statistically significant.

## Results

Table S5 shows the experimental groups, number of rats, and corresponding mortality rates. In the present study, except for 25 rats with bleeding less than 8 points excluded, a total of 392 rats were used (Sham group: 73 rats, SAH groups: 319 rats). The overall mortality rate in the SAH group was 18.18 ​% (58/319). Typical SAH images are shown in Fig. S2. The degree of bleeding was not significantly different between the SAH groups (Fig. S4).

### RIG-I is activated in the ipsilateral cortex of SAH rats and hBMVECs induced by OxyHb

**Fig. 1:**
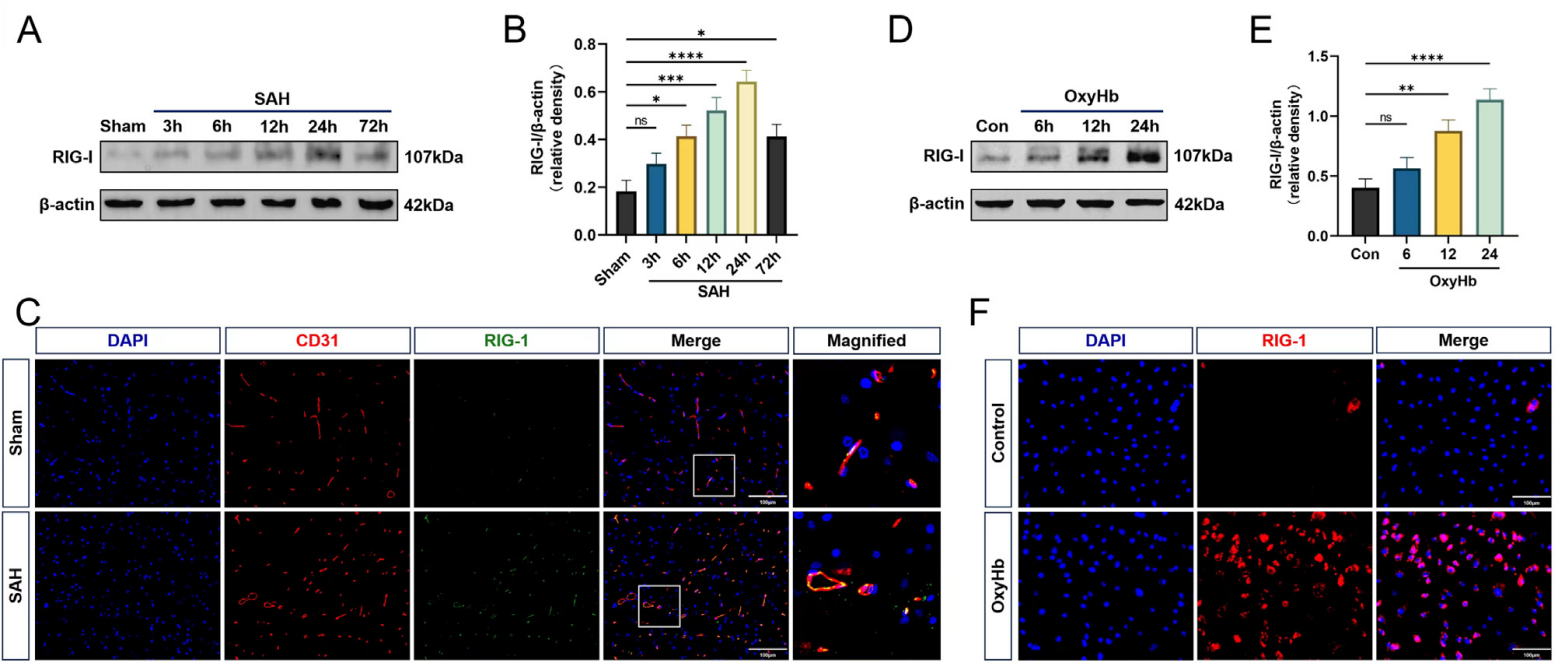
**The levels of RIG-1 in SAH rats and *in vitro* OxyHb-treated hBMVEC cells.** (A–B) Western blot showing RIG-1 expression at 3h, 6h, 12h, 24h, and 72h following SAH onset, n ​= ​6. (C) Co-staining of CD31 (red) and RIG-1 (green) was showed in endothelial cells at 24 ​h after SAH, n ​= ​3. (D–E) Western blot showing expression level of RIG-1 in OxyHb-treated hBMVEC cells, n ​= ​6/group. (F) The level of RIG-1 detected by immunofluorescence staining in OxyHb-treated hBMVEC cells, n ​= ​3/group. Scale bar ​= ​100 ​μm. Data was represented as mean ​± ​SD. ∗P ​< ​0.05, ∗∗P ​< ​0.01, ∗∗∗P ​< ​0.001, ∗∗∗∗P ​< ​0.0001 vs Sham group. ns, not significant.

Western blot was used to determine the protein level of RIG-1 after SAH. RIG-I was activated 6 ​h after SAH, peaked 24 ​h after SAH, and then declined 72 ​h after SAH (Fig. 1A and B). In the *in vitro* SAH model simulated using OxyHb stimulation of hBMVECs, Western blot results showed that the expression level of RIG-1 in hBMVECs increased significantly after 12 ​h of incubation with OxyHb and continued to increase at 24 ​h (Fig. 1D and E). In addition, double immunofluorescence labeling of RIG-I revealed that the number of RIG-I-positive BMVECs increased in SAH rats (24 ​h) compared to that in the sham group (Fig. 1C). Similarly, OxyHb incubation enhanced the RIG-I immunostaining signal in hBMVECs (Fig. 1F).

### Endogenous RIG-I knockdown attenuates short- and long-term neurological dysfunction, brain edema, histological damage, and neuronal degeneration in SAH rats

**Fig. 2:**
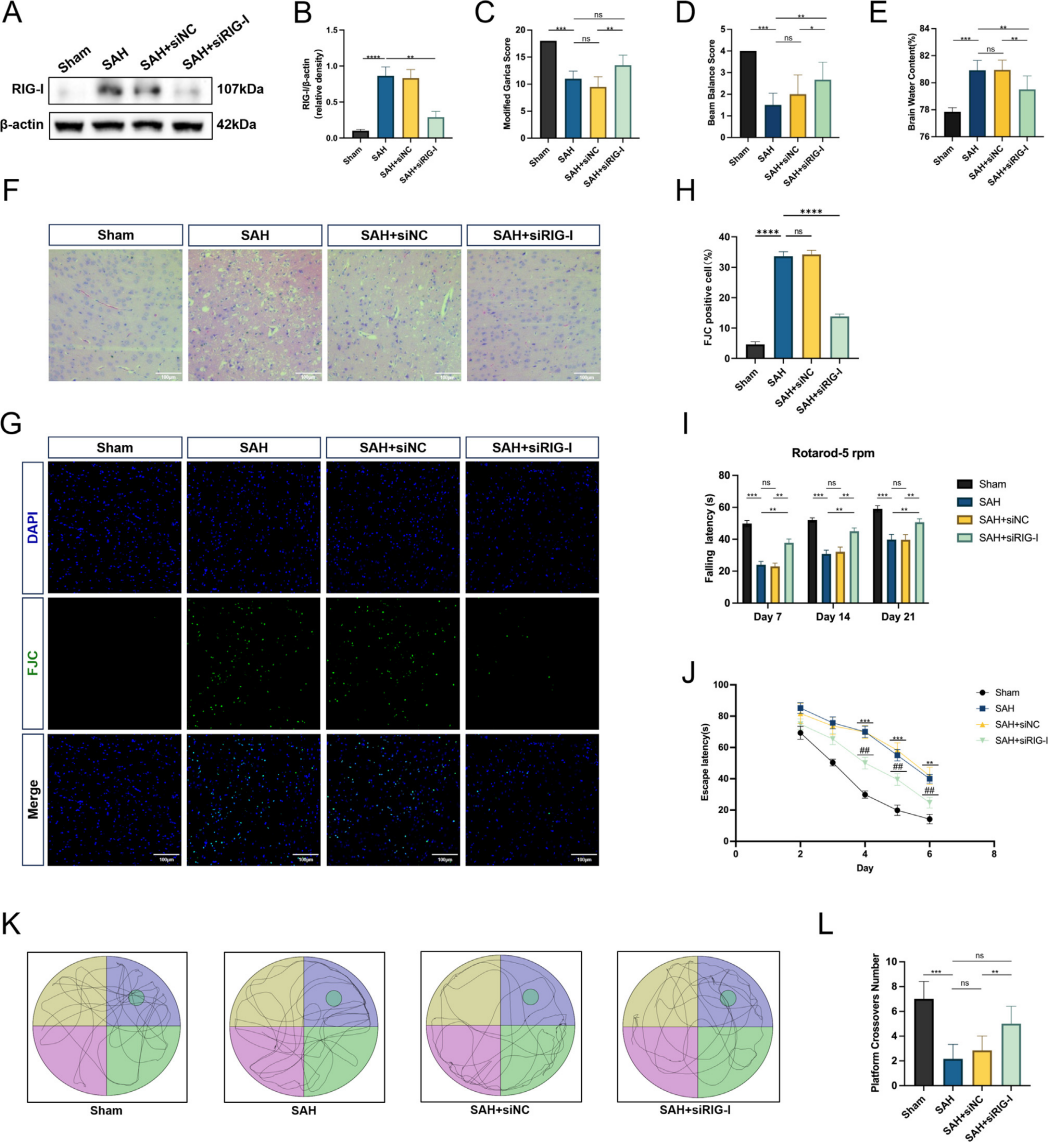
**Endogenous knockdown of RIG-I attenuates short-term & long-term neurological dysfunction, brain edema, histological damage and neuronal degeneration in SAH rats.** (A–B) Representative Western blot images of endogenous knockdown of RIG-I in SAH rat brain and its quantitative results. (C) The result of Modified Garcia score. n ​= ​6. (D) The result of Beam Balance score. n ​= ​6. (E) The result of brain water content. n ​= ​6. (F) Representative photographs of hematoxylin and eosin-stained sections of temporal cortex. Scale bar ​= ​100 ​μm (G–H) Representative observations of immunofluorescence analysis and quantification of FJC-positive cells. Scale bar ​= ​100 ​μm (I) 5-rpm rotarod test on days 7, 14, and 21 after SAH. n ​= ​10. (J) Escape latency in the hidden platform trial of the water maze test. n ​= ​10. ∗∗P ​< ​0.01 and ∗∗∗P ​< ​0.001 vs. Sham group and ##P ​< ​0.01 vs. SAH group. (K–L) The number of times each rat crossed the original position of the platform in the probe trial of the water maze test. n ​= ​10. ∗: P ​< ​0.05, ∗∗: P ​< ​0.01, ∗∗∗: P ​< ​0.001, and ∗∗∗∗: P ​< ​0.0001. ns, not significant.

To further verify the role of RIG-I after SAH, siRNA was used to endogenously knock down RIG-I expression after SAH (Fig. 2A and B). Based on the evaluation of the modified Garcia and beam balance neurological scores assessed 24 ​h after the SAH model establishment, RIG-I knockdown significantly reversed the short-term neurological dysfunction caused by SAH (Fig. 2C and D). The brain water content in rats was measured to detect brain edema. The SAH group had significant brain edema compared to that in the Sham group, and RIG-I knockdown reduced the brain edema caused by SAH (Fig. 2E). Similarly, H&E staining results showed that SAH caused severe histological damage to the temporal lobe of rats, while RIG-I knockdown alleviated this damage (Fig. 2F). In addition, neuronal degeneration occurred after SAH, and endogenous reduction in RIG-I also decreased the proportion of FJC-positive cells (Fig. 2G and H).

To assess the sensorimotor coordination and balance abilities of rats, rotarod tests were performed at 5-rpm and 10-rpm to evaluate the animals. The fall latency of rats after SAH was significantly shorter than that of the sham group. The performance of the SAH ​+ ​siNC group was similar to that of the SAH group. However, the fall latency of rats with endogenous RIG-I knockdown after SAH was significantly longer than that of the SAH&SAH ​+ ​siNC groups (Fig. 2I & S5). The water maze test is a commonly used method to evaluate the long-term spatial memory ability of rats after SAH. There was no significant difference in escape latency among the four groups in the initial visible platform test (Fig. S6A). Subsequently, the escape latency of the SAH and SAH ​+ ​siNC groups was significantly longer than that of the Sham group on days 4–6 of the training period, while the escape latency of the SAH ​+ ​siRIG-I group was shorter than that of the above two groups (Fig. 2J). In addition, the number of times rats in the SAH and SAH ​+ ​siNC groups crossed the original position of the platform in the probe trial was significantly lower than that in the Sham group, but the damage in rats with RIG-I knockdown before SAH was not severe (Fig. 2K and L). It is worth mentioning that there was no statistical difference in the swimming speed and distance of the four rat groups (Figs. S6B and C). However, it is worth noting that although there were changes in the neurological function of the rats, there was no statistically significant difference in the mortality rate among the SAH groups in Experiment 2 (Fig. S7).

### Rats with endogenous RIG-I knockdown have less BBB damage after SAH

**Fig. 3:**
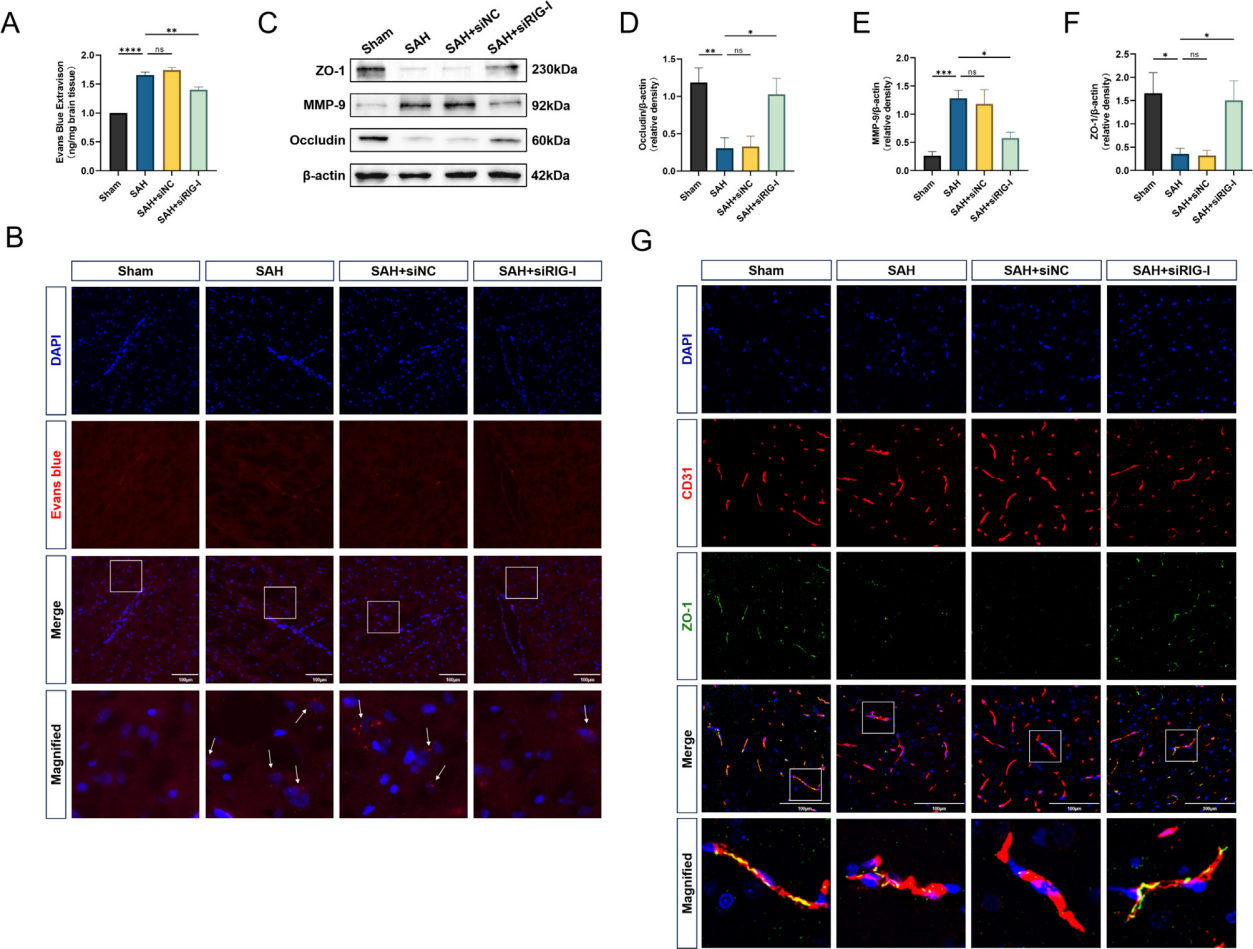
**Rats with endogenous knockdown of RIG-I have less severe blood-brain barrier damage after SAH.** (A) The result of Evans blue extravasation level 24 ​h after SAH, n ​= ​6. (B). Representative images of Evans blue spontaneous fluorescence, n ​= ​3. (C–F) Representative Western blot images and densitometric quantification of the expression of ZO-1, MMP-9, Occludin 24 ​h after SAH, n ​= ​6. (G) Representative double-immunofluorescence staining of ZO-1 (green) and endothelial cells (CD31, red) in the ipsilateral cortex. Scale bar ​= ​100 ​μm ∗: P ​< ​0.05, ∗∗: P ​< ​0.01, ∗∗∗: P ​< ​0.001, and ∗∗∗∗: P ​< ​0.0001. ns, not significant.

The level of Evans blue extravasation after SAH was used to assess BBB permeability. The extravasation level in the SAH ​+ ​siNC group was close to that in the SAH group, while the level in the SAH ​+ ​si-RIG-I group was significantly lower than that in the SAH group (Fig. 3A). In addition, we also detected the spontaneous fluorescence of Evans blue, which showed that there was no extravasation of point like fluorescence around cerebral microvessels in the sham group (Fig. 3B). However, significant point like fluorescence was observed in both the SAH group and SAH ​+ ​siNC group, while the infiltration of point like fluorescence was significantly reduced in the SAH ​+ ​siRIG-I group, indicating an improvement in BBB leakage. Western blot was used to determine the expression levels of cytokines related to BBB function. The expression levels of ZO-1, MMP-9, and occludin in the SAH group were significantly lower than those in the Sham group. The above protein levels in the SAH ​+ ​siNC group were similar to those in the SAH group, while the protein levels in the SAH ​+ ​siRIG-I group were higher than those in the SAH group (Fig. 3C–F). More intuitively, the fluorescence intensity of tight junction protein ZO-1 in the SAH and SAH ​+ ​siNC groups was significantly lower than that in the Sham group, while fluorescence intensity in the SAH ​+ ​siRIG-I group was between those observed in the Sham and SAH groups (Fig. 3G).

### Rats with endogenous RIG-I knockdown have less neuroinflammation after SAH

**Fig. 4:**
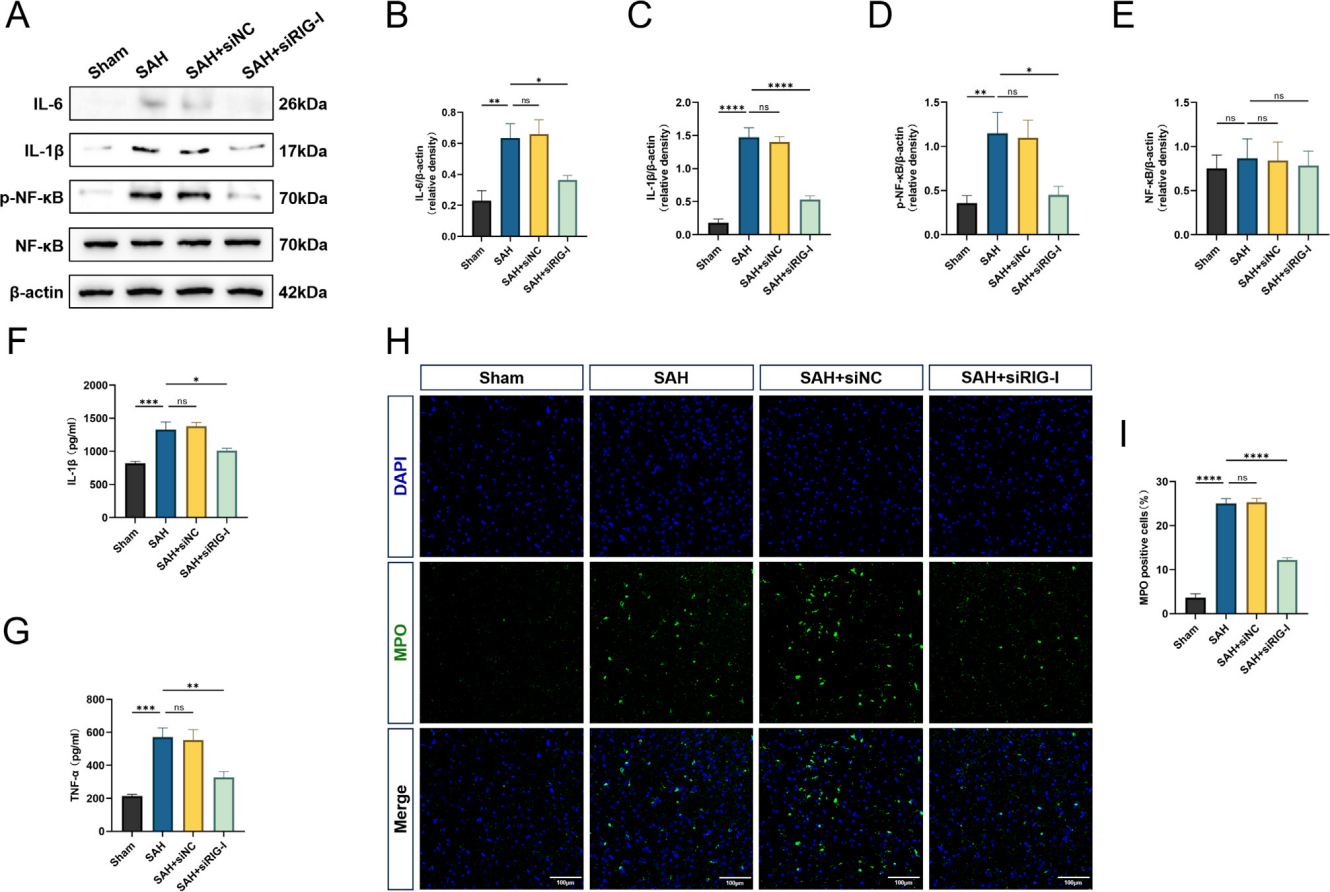
**Rats with endogenous knockdown of RIG-I have less neuroinflammation after SAH.** (A–E) Representative Western blot images and densitometric quantification of the expression of IL-6, IL-1β, *p*-NFκB, NFκB 24 ​h after SAH, n ​= ​6. (F–G) Concentrations of IL-1β and TNF-α in ipsilateral cortical tissue after homogenization measured by ELISA, n ​= ​6. (H–I) Representative observations of immunofluorescence analysis and quantification of MPO-positive cells. Scale bar ​= ​100 ​μm ∗: P ​< ​0.05, ∗∗: P ​< ​0.01, ∗∗∗: P ​< ​0.001, and ∗∗∗∗: P ​< ​0.0001. ns, not significant.

To investigate the role of RIG-I in neuroinflammation, the levels of a series of inflammatory factors and neutrophil infiltration were examined. Western blot results showed that the levels of typical inflammatory factors IL-6, IL-1β, and *p*-NFκB were higher in the SAH group than those in the Sham group. The above protein levels in the SAH ​+ ​siNC group were close to those in the SAH group, while the levels in the SAH ​+ ​siRIG-I group were significantly lower than those in the SAH group (Fig. 4A–D). Total NF-κB protein levels remained equal across groups (Fig. 4A–E). Similarly, the ELISA results of brain tissue homogenates also showed a similar trend. The levels of TNF-α and IL-1β in the SAH and SAH ​+ ​siNC groups were significantly higher than those in the Sham group, while the levels in the SAH ​+ ​siRIG-I group were lower than those in the SAH group (Fig. 4F and G). Finally, SAH induced a significant increase in neutrophil-derived myeloperoxidase (MPO) in the ipsilateral hemisphere, and the SAH ​+ ​siNC group was similar to the SAH group, while the level of neutrophil accumulation in the SAH ​+ ​siRIG-I group was lower (Fig. 4H and I).

### Rats with endogenous RIG-I knockdown have milder BMVECs pyroptosis after SAH

**Fig. 5:**
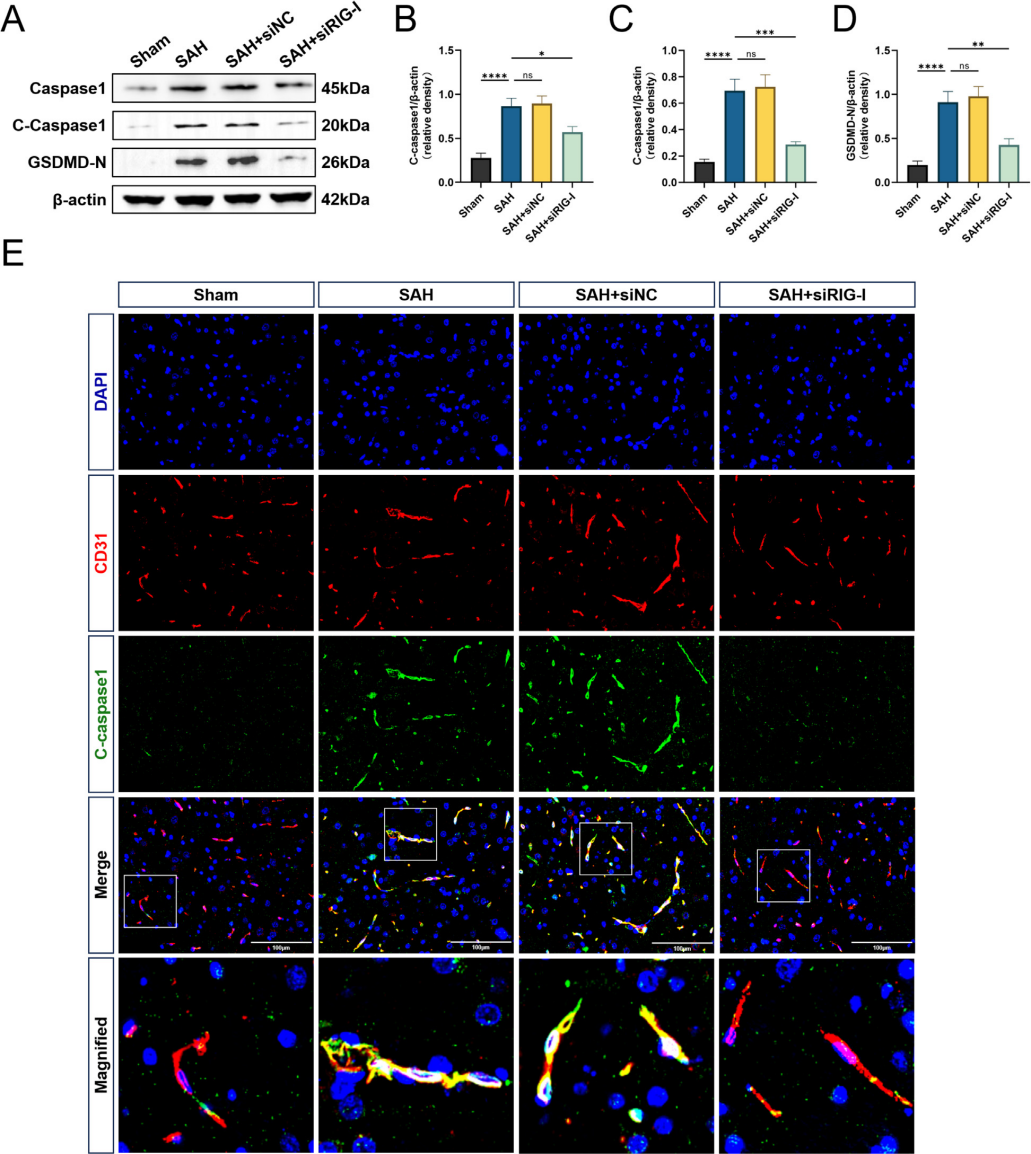
**Rats with endogenous knockout of RIG-I have less endothelial cell pyroptosis after SAH.** (A–D) Representative Western blot images and densitometric quantification of the expression of Caspase-1, Cleaved Caspase-1, GSDMD-N 24 ​h after SAH, n ​= ​6. (E) Representative double-immunofluorescence staining of Cleaved Caspase-1 (green) and endothelial cells (CD31, red) in the ipsilateral cortex. Scale bar ​= ​100 ​μm ∗: P ​< ​0.05, ∗∗: P ​< ​0.01, ∗∗∗: P ​< ​0.001, and ∗∗∗∗: P ​< ​0.0001. ns, not significant.

Pyroptosis is one of the causes of neuroinflammation. The role of RIG-I in pyroptosis after SAH was examined. The Western blot results showed that the levels of pyroptosis-related proteins caspase-1, cleaved caspase-1, and GSDMD-N were higher in the SAH group than those in the Sham group. The above protein levels in the SAH ​+ ​siNC group were close to those in the SAH group, while the levels in the SAH ​+ ​siRIG-I group were significantly lower than those in the SAH group (Fig. 5A–D). Double immunofluorescence staining was used to identify pyroptosis in BMVECs. The results showed that cleaved caspase-1 levels increased after SAH and co-localized with the BMVECs marker CD31. The SAH ​+ ​siNC group levels were not different from those in the SAH group, while cleaved caspase-1 levels in the SAH ​+ ​siRIG-I group were lower than those in the SAH group.

### RIG-I knockdown protects hBMVECs from OxyHb-induced cell dysfunction

**Fig. 6:**
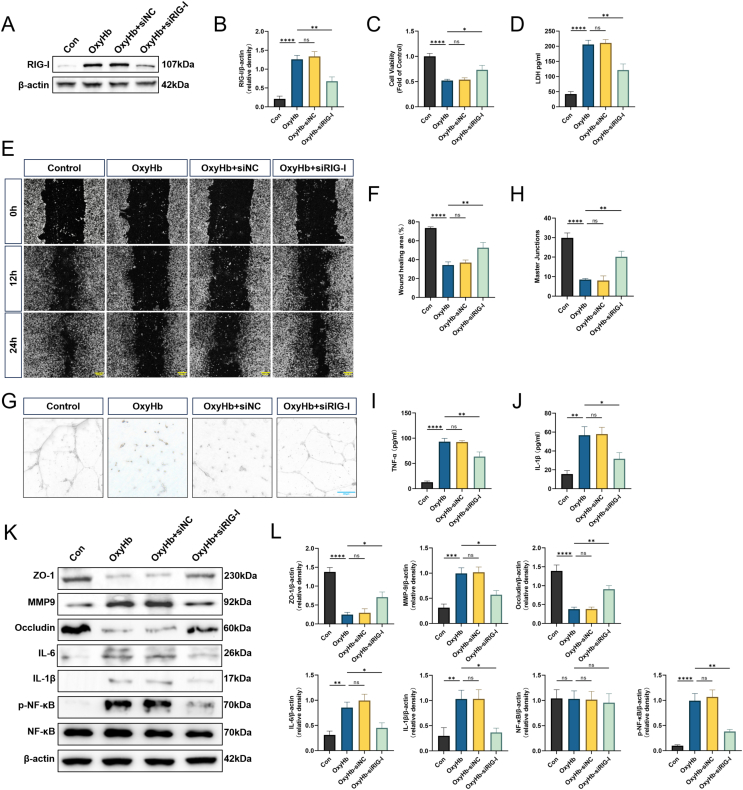
**RIG-I knockdown protects hBMVECs from OxyHb-induced cell dysfunction.** (A–B) Representative Western blot images of knockdown of RIG-I in Oxyhemoglobin (OxyHb) induced hBMVEC and its quantitative results. (C) Quantitative analysis of cell viability detected by MTT assays. n ​= ​6. (D) Quantitative analysis of cytotoxicity was evaluated with the LDH assay. n ​= ​6. (E–F) Representative images and quantitative analysis of migration distance to evaluate the migration ability of hBMVEC cells by wound healing assay. n ​= ​6. Scale bar ​= ​200 ​μm. (G–H) Evaluation of the tube-forming ability of hBMVEC and quantitative analysis results. n ​= ​6. Scale bar ​= ​200 ​μm. (I–J) Concentrations of TNF-α and IL-1β in cell culture supernatant measured by ELISA. n ​= ​6. (K–L) Representative Western blot images and densitometric quantification of the expression of ZO-1, MMP-9, Occludin, IL-6, IL-1β, *p*-NFκB, NFκB 24 ​h after OxyHb induced SAH in-virto model. n ​= ​6. ∗: P ​< ​0.05, ∗∗: P ​< ​0.01, ∗∗∗: P ​< ​0.001, and ∗∗∗∗: P ​< ​0.0001. ns, not significant.

In the SAH *in vitro* model established by stimulating hBMVECs with OxyHb, Western blot results showed that RIG-I expression was successfully knocked down using siRNA (Fig. 6A and B). MTT and LDH assays were used to assess cell viability and cytotoxicity, respectively. Cell viability decreased significantly and cytotoxicity increased after the OxyHb treatment. The performance of the siNC-applied group was similar to that of the OxyHb group. However, cell viability of the RIG-I knockdown group was higher than that of the OxyHb group, while the toxicity was lower than that of the OxyHb group (Fig. 6C and D). Cell scratch and tube formation assays were used to evaluate the cellular function of hBMVECs. Cell migration ability of the OxyHb and OxyHb ​+ ​siNC groups was significantly weaker than that of the Control group, while cell migration ability was restored in the OxyHb ​+ ​siRIG-I group (Fig. 6E and F). Similarly, the tube-forming ability of hBMVECs after OxyHb treatment was reduced compared to that in the Control group and junction formation was difficult on Matrigel. The same was true for the OxyHb ​+ ​siNC group, although more junctions were observed in the OxyHb ​+ ​siRIG-I group than in the above two groups (Fig. 6G and H). Cell culture supernatants were used to measure inflammation levels. The TNF-α and IL-1β levels increased after the OxyHb treatment. The siNC group results were similar to those of the OxyHb group, while the levels of the siRIG-I group decreased compared to those of the previous two groups (Fig. 6I and J). Western blot results showed that OxyHb eliminated the expression of ZO-1 and occludin in hBMVECs and increased the expression of inflammatory factors, such as MMP-9, IL-6, IL-1β, and *p*-NFκB, while the total NFκB content did not change. The OxyHb ​+ ​siNC group results were consistent with those of the OxyHb group. In the group where RIG-I was knocked-down using siRNA and OxyHb treatment was administered, ZO-1 and occludin expression levels were restored, MMP-9, IL-6, IL-1β, and *p*-NFκB levels were decreased, and total NFκB content remained consistent with that observed in the other groups (Fig. 6K and L).

### RIG-I knockdown alleviates caspase-1-mediated pyroptosis in hBMVECs treated with OxyHb

**Fig. 7:**
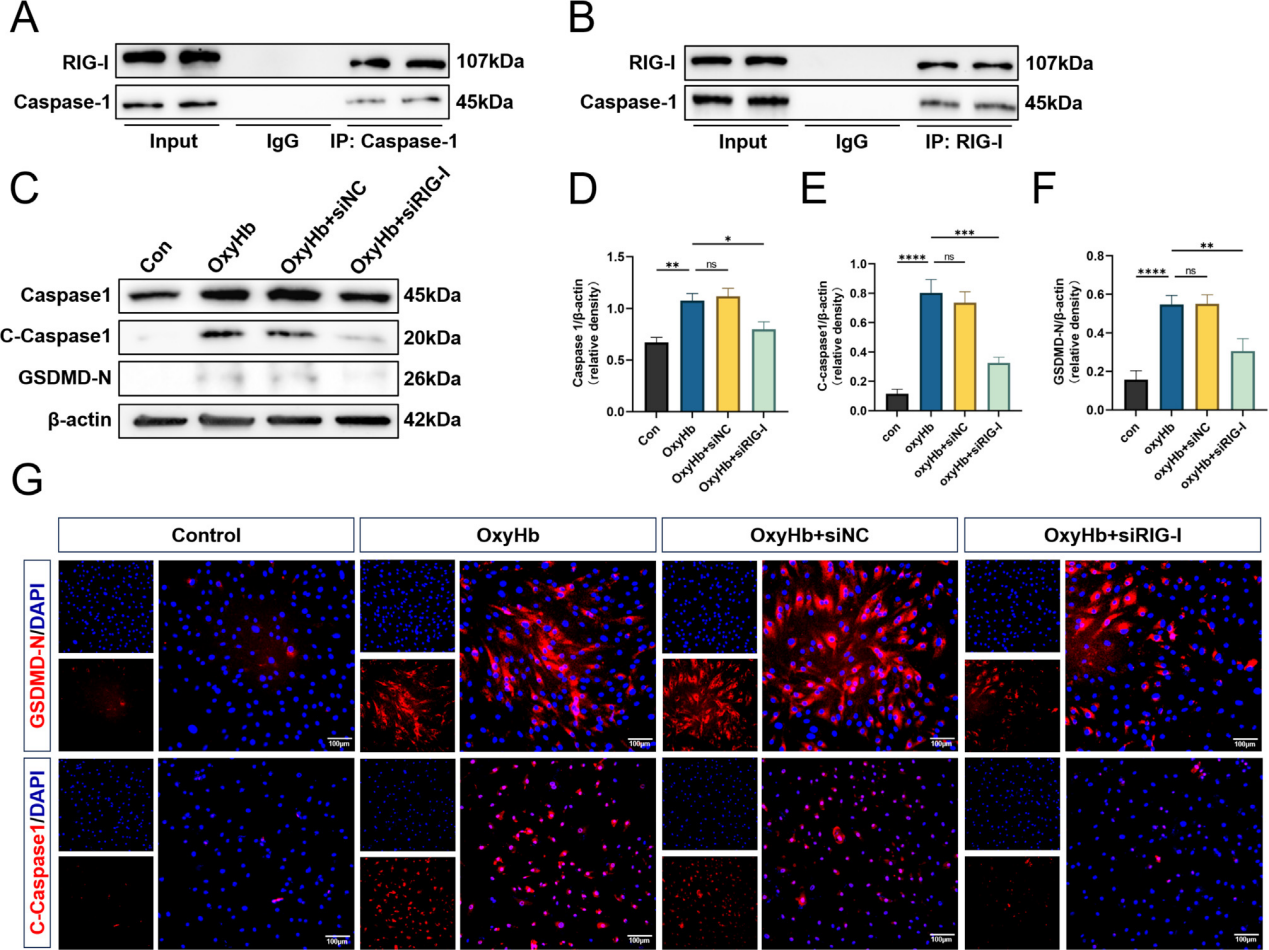
**RIG-I knockdown alleviates Caspase-1-mediated pyroptosis in hBMVEC treated with OxyHb.** (A–B) The interaction of Caspase-1 and RIG-I was tested by CO-IP in OxyHb-treated hBMVEC. (C–F) Representative Western blot images and densitometric quantification of the expression of Caspase-1, Cleaved Caspase-1, GSDMD-N 24 ​h after OxyHb induced SAH in-virto model. n ​= ​6. (G) The level of Cleaved Caspase-1 and GSDMD-N detected by immunofluorescence staining in hBMVEC. Scale bar ​= ​100 ​μm∗: P ​< ​0.05, ∗∗: P ​< ​0.01, ∗∗∗: P ​< ​0.001, and ∗∗∗∗: P ​< ​0.0001. ns, not significant.

RIG-I has been shown to activate caspase-1-mediated pyroptosis in other diseases [23]. The Co-IP method was used to identify the interaction between RIG-I and caspase-1 in order to further confirm the mechanism of RIG-I involvement in BMVECs inflammation and pyroptosis. The results showed that RIG-I was directly related to caspase-1, indicating that RIG-I regulated caspase-1 activation (Fig. 7A and B). Western blot was subsequently used to determine pyroptosis-related protein levels. The results showed that RIG-I knockdown significantly reduced the expression levels of caspase-1, cleaved caspase-1, and GSDMD-N in hBMVECs treated with OxyHb (Fig. 7C–F). Immunofluorescence staining demonstrated that GSDMD-N and cleaved caspase-1 expression was upregulated in hBMVECs treated with OxyHb *in vitro* compared to that in the control group, while RIG-I knockdown significantly reduced the expression of both (Fig. 7G).

### Caspase-1 inhibitor VX-765 reverses tight junction loss, inflammation, and pyroptosis in an in vitro SAH model exacerbated by RIG-I activation

**Fig. 8:**
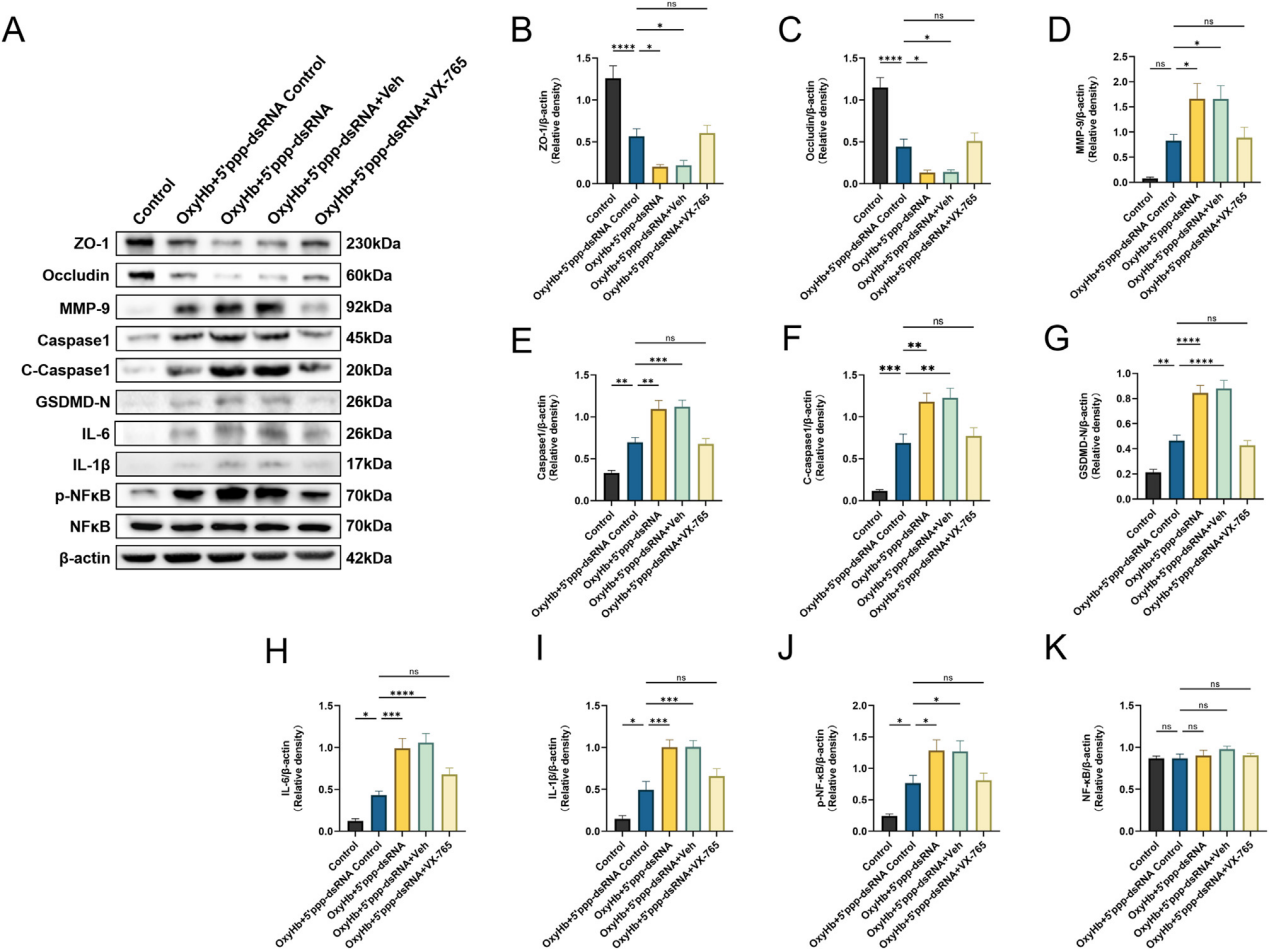
**Caspase-1 inhibitor VX-765 reverses tight junction loss, inflammation, and pyroptosis in an *in-vitro* SAH model exacerbated by RIG-1 activation.** (A–L) Representative Western blot images and densitometric quantification of the expression of RIG-I, ZO-1, Occludin, MMP-9, Caspase-1, Cleaved Caspase-1, GSDMD-N, IL-6, IL-1β, *p*-NFκB, NFκB 24 ​h after OxyHb induced SAH in-virto model, n ​= ​6. ∗: P ​< ​0.05, ∗∗: P ​< ​0.01, ∗∗∗: P ​< ​0.001, and ∗∗∗∗: P ​< ​0.0001. ns, not significant.

To further verify the relationship between RIG-I and caspase-1, RIG-I was activated *in vitro* by applying 5′ppp-dsRNA (Fig. S8). Western blot results showed that 5′ppp-dsRNA further exacerbated the loss of ZO-1 and occludin and increased MMP-9, caspase-1, cleaved caspase-1, GSDMD- N, IL-6, IL-1β, and *p*-NFκB expression levels compared to those in the OxyHb+5′ppp-dsRNA control group. In addition, 10-μM caspase-1 inhibitor VX-765 alleviated tight junction loss, reduced MMP-9, caspase-1, cleaved caspase-1, GSDMD, IL-6, IL-1β, and *p*-NFκB levels, and increased other inflammatory and pyroptotic cytokine levels, while the total NFκB level remained consistent in each group (Fig. 8A–K).

### Caspase-1 inhibitor VX-765 reverses early brain injury in SAH rats with aggravated injury after RIG-I activation

To further validate the involvement of RIG-I in the mechanism of EBI, we overexpressed RIG-I using its activator, 5′ppp-dsRNA. Western blot results demonstrated that RIG-I was activated in rat brain tissue following SAH. Intraventricular injection of 5 ​μg/kg of 5′ppp-dsRNA was sufficient to further elevate RIG-I expression, showing statistically significant differences compared to the SAH group (P ​< ​0.01). However, the effects of 10 ​μg/kg and 20 ​μg/kg of 5′ppp-dsRNA on further activating RIG-I were similar to that of 5 ​μg/kg, with no statistically significant differences (Fig. S9). Subsequently, a dosage of 5 ​μg/kg was selected for further research.

**Fig. 9:**
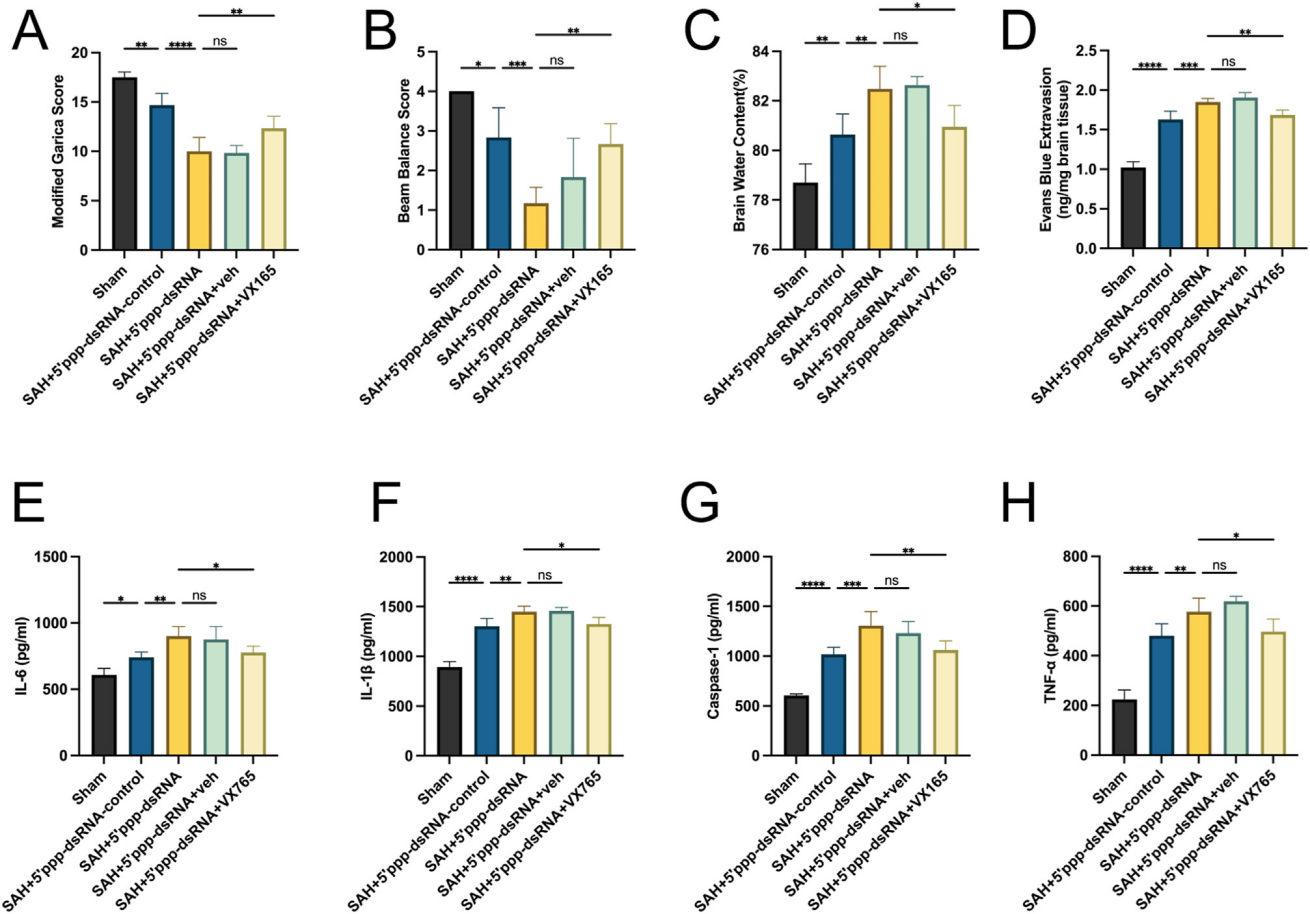
**Caspase-1 inhibitor VX-765 reverses early brain injury in SAH rats with aggravated injury after RIG-I activation.** (A) The result of Modified Garcia score, n ​= ​6. (B) The result of Beam Balance score, n ​= ​6. (C) The result of brain water content, n ​= ​6. (D) The result of Evans blue extravasation level 24 ​h after SAH, n ​= ​6. (E–H) Concentrations of IL-6, IL-1β, Caspase-1 and TNF-α in ipsilateral cortical tissue after homogenization measured by ELISA, n ​= ​6.

Subsequently, we applied the Caspase-1 inhibitor VX765 to further investigate the mechanism by which RIG-I is involved in EBI. The results showed no significant differences in bleeding volume among the groups 24 ​h post-SAH, and no differences in mortality rates were observed among the SAH groups (Fig. S10-11). The modified Garcia score in the SAH+5′ppp-dsRNA control group was lower than that in the Sham group (P ​< ​0.01), while the score in the SAH+5′ppp-dsRNA group decreased further (P ​< ​0.0001 vs. SAH+5′ppp-dsRNA control group). There was no statistically significant difference in the score between the SAH+5′ppp-dsRNA ​+ ​vehicle group and the SAH+5′ppp-dsRNA group. However, the SAH+5′ppp-dsRNA ​+ ​VX765 group reversed the decreasing trend in the score, which was higher than that in the SAH+5′ppp-dsRNA control group (P ​< ​0.01) (Fig. 9A). The results of the balance beam experiment also followed a similar trend to the modified Garcia scores. The scores in the SAH+5′ppp-dsRNA control group were lower than those in the Sham group (P ​< ​0.01), and further decreased in the SAH+5′ppp-dsRNA group (P ​< ​0.01 vs. SAH+5′ppp-dsRNA control group). There was no significant difference in scores between the SAH+5′ppp-dsRNA ​+ ​solvent group and the SAH+5′ppp-dsRNA group. However, the scores in the SAH+5′ppp-dsRNA ​+ ​VX765 group were higher than those in the SAH+5′ppp-dsRNA control group (P ​< ​0.05).The results indicate that the RIG-I activator 5′ppp-dsRNA exacerbates neurological dysfunction following SAH, whereas the Caspase-1 inhibitor VX765 reverses this exacerbation (Fig. 9B).

The brain edema level and Evans blue permeability test were used to assess the degree of brain edema and BBB leakage, respectively. The results showed that the brain edema in the SAH+5′ppp-dsRNA control group was more severe than that in the Sham group (P ​< ​0.01). The SAH+5′ppp-dsRNA group further aggravated the brain edema (P ​< ​0.05 vs. SAH+5′ppp-dsRNA control group), while no significant difference in brain edema was observed between the SAH+5′ppp-dsRNA ​+ ​solvent group and the SAH+5′ppp-dsRNA group. However, the edema was alleviated in the SAH+5′ppp-dsRNA ​+ ​VX765 group, which was lower than the SAH+5′ppp-dsRNA control group (P ​< ​0.05) (Fig. 9C). Compared to the Sham group, the Evans blue extravasation was significantly increased in the SAH+5′ppp-dsRNA control group (P ​< ​0.05). The SAH+5′ppp-dsRNA group further increased the extravasation (P ​< ​0.05 vs. SAH+5′ppp-dsRNA control group). No significant difference in extravasation was found between the SAH+5′ppp-dsRNA ​+ ​solvent group and the SAH+5′ppp-dsRNA group, while the SAH+5′ppp-dsRNA ​+ ​VX765 group reversed the increase in Evans blue extravasation (P ​< ​0.05 vs. SAH+5′ppp-dsRNA group) (Fig. 9D). These results indicate that 5′ppp-dsRNA can further exacerbate brain edema and BBB leakage after SAH, while VX765 reverses this trend.

Next, we used ELISA to evaluate the levels of neuroinflammatory and pyroptosis factors in the rat brain tissue lysate, including IL-1β, IL-6, TNF-α, Caspase-1, and Cleaved-caspase-1 (Fig. 9E–H). The results showed that in the SAH+5′ppp-dsRNA control group, IL-1β, IL-6, TNF-α, and Caspase-1 levels were higher than in the Sham group, while the SAH+5′ppp-dsRNA group further increased the levels of these factors. This suggests that 5′ppp-dsRNA can exacerbate neuroinflammation and pyroptosis after SAH. No significant difference in cytokine levels was observed between the SAH+5′ppp-dsRNA ​+ ​solvent group and the SAH+5′ppp-dsRNA group, but the cytokine levels in the SAH+5′ppp-dsRNA ​+ ​VX765 group were lower than those in the SAH+5′ppp-dsRNA control group. This indicates that inhibiting Caspase-1 can counteract the neuroinflammation and pyroptosis induced by RIG-I activation.

### Correlation between RIG-I and caspase-1 and IL-1β levels and outcomes in CSF patients

**Fig. 10:**
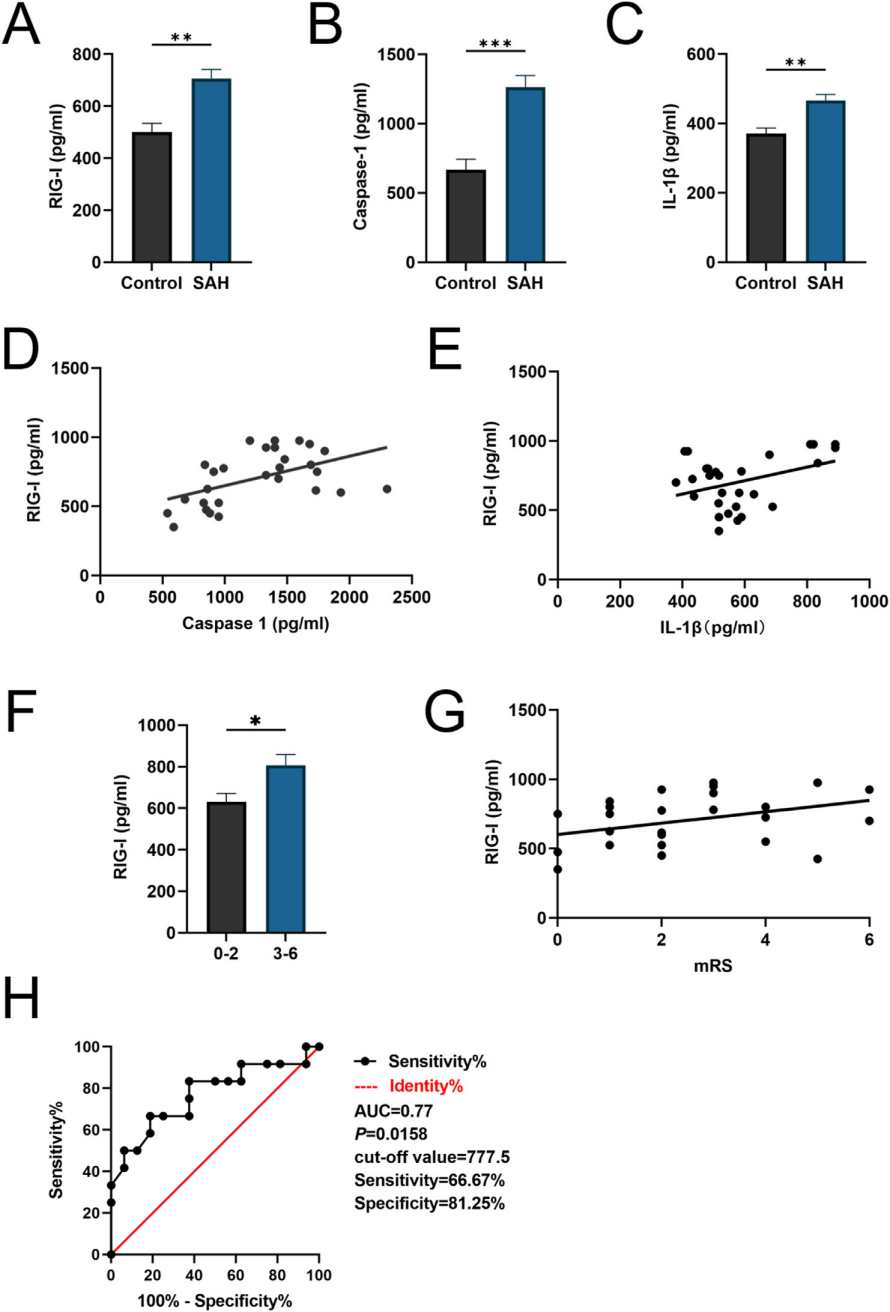
**The expression of RIG-I, Caspase-1, and IL-1β in human CSF and the correlation between RIG-I and Caspase-1, IL-1β, and outcome.** (A–C) The levels of RIG-I, Caspase-1 and IL-1β in human CSF were detected by ELISA assay in control (n ​= ​12) and SAH (n ​= ​28) patients. (D–E) The linear-regression analysis showed the relationship between RIG-I and Caspase-1/IL-1β. (F) ELISA assay for the RIG-I in CSF from SAH patients with different mRS scores (0–2 vs 3–6). (G) The linear-regression analysis showed the relationship between RIG-I and mRS scores. (H) ROC showed that RIG-I levels in human CSF after SAH have a moderate diagnostic value for outcome at 6-month follow-up. Data was represented as mean ​± ​SD. ∗P ​< ​0.05, ∗∗P ​< ​0.01, ∗∗∗P ​< ​0.001 vs control group.

To determine the levels of RIG-I, caspase-1, and IL-1β, the CSF of SAH patients and healthy control group was analyzed and ELISA was performed. In this study, a total of 40 patients were included, with an average age of 55.65 ​± ​8.52 years, comprising 24 males and 16 females. Among them, 28 patients were in the SAH group, aged 56.18 ​± ​8.42 years, including 18 males and 10 females. The 12 patients in the healthy control group had an average age of 54.42 ​± ​8.99 years, with 6 males and 6 females. There were no statistically significant differences in age or gender distribution between the two groups. RIG-I, caspase-1, and IL-1β levels were significantly increased in the SAH group compared to those in the control group (Fig. 10A–C). Pearson's correlation coefficient was subsequently utilized to analyze the relationship between RIG-I levels and caspase-1 and IL-1β levels in addition to patients' mRS scores 6 months after discharge. The results revealed that RIG-I was positively correlated with caspase-1 (r ​= ​0.5033, *P* ​= ​0.0063) and IL-1β (r ​= ​0.3774, *P* ​= ​0.0477; Fig. 10D and E). RIG-I level was higher in the group with poor six-month outcomes (mRS: 3–6) than in the group with good outcomes (mRS: 0–2; Fig. 10F). RIG-I level was also positively correlated with the mRS score at six months (r ​= ​0.3827, *P* ​= ​0.0444; Fig. 10G). Receiver operating characteristic curve analysis showed that post-SAH RIG-I levels (Area under the curve [AUC] ​= ​0.77, sensitivity ​= ​66.67 ​%, specificity ​= ​81.25 ​%, *P* ​= ​0.0158) were moderately diagnostic for six-month mRS, with RIG-I cut-off value of 777.5 ​pg/mL (Fig. 9H).

## Discussion

The aim of the present study was to explore the mechanism of classical immune signaling receptor RIG-I involvement in SAH-EBI. The research results were as follows: (1) RIG-I was upregulated in a time-dependent manner in both *in vivo* and *in vitro* SAH models and reached its peak 24 ​h after modeling. Immunofluorescence staining showed a significant increase in RIG-I level in BMVECs. (2) Rats with endogenous RIG-I knockdown using siRNA showed mild short- and long-term neurological function, brain edema, and neuronal damage after SAH. (3) Endogenous RIG-I knockdown alleviated BMVECs pyroptosis, BBB damage, and neuroinflammation after SAH. (4) In hBMVECs stimulated by OxyHb, RIG-I knockdown increased cell survival rate, reduced cell functional damage and inflammation, and alleviated caspase-1-mediated cell pyroptosis, while caspase-1 inhibition mitigated pyroptosis exacerbation caused by RIG-I activation. In addition, RIG-I levels were elevated in the CSF of SAH patients and were correlated with caspase-1 and IL-1 β levels and six-month outcome.

The present study results indicated that RIG-I was localized in BMVECs and its levels increased after SAH. RIG-I directly activated caspase-1-mediated BMVECs pyroptosis and participated in BBB damage and neuroinflammation after SAH, which had adverse effects on SAH-EBI.

Hemorrhagic blood and hemoglobin breakdown products infiltrate the subarachnoid space after SAH occurrence, leading to a pathological cascade reaction in the brain and causing a series of secondary injuries, including microcirculation dysfunction, BBB damage, neuroinflammation, oxidative stress, brain edema, and abnormal cell death [5]. Cellular pyroptosis usually manifests as continuous swelling of cells until the cell membrane ruptures, leading to the release of a large amount of cellular contents and pro-inflammatory cytokines, which in turn activates a strong inflammatory response, usually exacerbating the SAH-EBI [42]. At present, it is widely believed that the classical cell pyroptosis pathway is mediated by caspase-1 and is triggered by a series of pattern recognition receptors that can form inflammasomes [43]. Moreover, caspase-1 activity is elevated in the CSF of SAH patients, and the higher the activity the worse the functional outcomes [44]. Similarly, the products of red blood cell lysis during SAH-EBI, including methemoglobin and heme, can stimulate BMVECs and induce an inflammatory cascade to promote neuroinflammation, which is one of the most important factors affecting SAH patient outcomes [45].

There have been many studies on glial cell and neuronal pyroptosis after SAH, but the endothelium is also considered to be a type of immune cell population that serves as a physical barrier between circulating blood components and the blood vessel wall, but the role of BMVECs pyroptosis in SAH has been rarely explored [46]. Recently, immunofluorescence staining experiments in a study by Hu et al. showed that cleaved caspase-1 level increased after SAH and co-localized with the BMVECs marker CD31, indicating that BMVECs also undergo pyroptosis after SAH. The present study also made a similar discovery. In addition, pyroptosis markers caspase-1, cleaved caspase-1, and GSDMD-N levels in hBMVECs stimulated by OxyHb increased.

RIG-I is a pattern recognition receptor that can identify common molecular features in dsRNA and ssRNA viral pathogens, playing a crucial role in innate antiviral immunity [47]. However, in recent years, it has been found that RIG-I can also recognize endogenous RNA, DNA, and protein, thus participating in inflammatory diseases, autoimmune diseases, atherosclerosis, and other diseases [48]. In the central nervous system, RIG-I has been found to promote immune response after ischemic stroke, and excessive immune response exacerbates NF-κB-mediated inflammation and brain damage [49]. The application of compounds, such as propyl gallate and saponins of *Panax notoginseng*, or Klotho overexpression can inhibit RIG-I-mediated neuroinflammation and alleviate brain damage [49]. However, there is currently no research evaluating whether RIG-I plays a role in SAH-EBI. In the current investigation, RIG-I expression was highly enhanced after SAH and RIG-I knockdown with siRNA alleviated neuroinflammation, brain edema, and neuronal damage after SAH, which is consistent with relevant reports on ischemic stroke [50]. In addition, RIG-I knockdown was accompanied by a significant reduction in cell apoptosis, and Co-IP results also confirmed that RIG-I directly bound to the pyroptosis switch caspase-1 to promote the expression of cleaved-caspase-1 and GSDMD-N and inflammatory cytokine IL-1β release. This phenomenon is consistent with viral diseases and macrophage damage caused by myoglobin, which is likely attributed to caspase-1 and RIG-I having structurally identical CARD regions [23]. The role of RIG-I in SAH is similar to the one described by Li et al. in crush syndrome-induced acute kidney injury (CS-AKI). They found that myoglobin promotes macrophage polarization to M1 type and pyroptosis via the RIG-I/caspase-1/GSDMD signaling pathway in CS-AKI [23]. However, the activating effect of myoglobin on RIG-I could not be clarified in the present study. Although hBMVECs stimulation with hemoglobin led to RIG-I activation, further *in vivo* experiments are still needed to determine which blood product caused RIG-I activation after SAH.

It is worth noting that RIG-I inhibition was accompanied by a reduction in BMVECs pyroptosis, thus playing a protective role against brain injury. Multiple studies in recent years have shown that RIG-I plays an important role in the physiological and pathological processes mediated by Endothelial cells. Asdonk et al. found that RIG-I activation can significantly impair endothelial-dependent mouse aorta vasodilation [51]. Ma et al. showed that a RIG-I signaling activator poly (I:C) inhibited VEGF-induced angiogenesis and vascular permeability *in vivo* and *in vitro* [52]. More importantly, RIG-I is an immune signal that is deeply involved in the inflammatory response of Endothelial cells, such as increasing COX-2 gene promoter activity to enhance COX-2-mediated inflammatory response or modulating the expression of two important inflammatory mediators IL-6 and IL-8 in senescent cells [53]. However, these studies were all based on peripheral Endothelial cells. Thus, how RIG-I activation in BMVECs after SAH specifically participates in the pathological process of the disease needs to be explored.

Endothelial cells are an important component of vascular units. However, most BMVECs are nonporous and can form a tight BBB compared to the peripheral vascular system, thereby limiting the entry of toxic components, blood cells, and pathogens into the brain [7]. Another BMVECs function is to promote information transmission between cells within the brain, such as neurons and glial cells, thus regulating nervous system damage [45]. Abnormal BMVECs function can exacerbate secondary damage in various central nervous system diseases [54,55]. Xu et al. found that BMVECs death caused by cell pyroptosis in ischemic brain injury leads to increased vascular permeability, BBB integrity disruption, and subsequent immune cell infiltration [55]. The present study found that RIG-I knockdown-mediated reduction in BMVECs pyroptosis was accompanied by BBB function recovery and decreased MPO infiltration. In addition, cellular pyroptosis was closely related to inflammatory response, leading to the release of cellular contents and the production of inflammatory cytokines, thereby exacerbating the inflammatory environment and worsening brain damage [55]. Moreover, alleviating OxyHb-mediated hBMVECs pyroptosis reduced the levels of pro-inflammatory factors IL-6, IL-1 β, and TNF-α. Therefore, there is reason to believe that reducing BMVECs pyroptosis can help alleviate the inflammatory environment in the brain after SAH. A reduction in BMVECs pyroptosis and neuroinflammatory response was observed after knocking down RIG-I *in vivo*, in addition to attenuating neurological function, brain edema, and BBB damage caused by SAH. However, how BMVECs pyroptosis regulates neuronal damage after SAH is still a topic worth exploring. Recently, Gu et al. found that BMVECs pyroptosis promotes microglial polarization and leads to neuronal apoptosis in intracerebral hemorrhage [56]. This is noteworthy for the present study not only because intracerebral hemorrhage and SAH have a similar pathogenesis, but also because hemin was used to stimulate ECs, which is similar to the present study's use of OxyHb to stimulate hBMVECs. Moreover, BMVECs were found to cause subsequent microglial polarization and neuronal apoptosis due to the release of IL-6 and IL-1β in the process of cell pyroptosis. More importantly, an increase in these inflammatory factors in hBMVECs treated with OxyHb was demonstrated, while RIG-I knockdown reduced their levels. Therefore, future research should explore their subsequent roles to clarify the relevant mechanisms.

The present research study had several limitations. First, NLRP3-induced cell pyroptosis is a key signaling pathway after SAH, and the relationship between RIG-I and NLRP3 was not explored [46]. Perhaps RIG-I is not associated with NLRP3 and can directly activate caspase-1, but this still needs to be verified in SAH. Second, the role of RIG-I was validated only in BMVECs. However, RIG-I is also widely present in other cells in the brain, such as astrocytes and microglia. The role of RIG-I in other cells and its involvement in SAH-EBI still need to be studied. Finally, although it was verified that RIG-I activated BMVECs pyroptosis and promoted neuroinflammation and neuronal damage, the specific mechanism of how pyroptosis induces inflammation and neuronal damage remains unclear and will be explored in future studies. In summary, the present study suggested that RIG-I promoted BBB damage and neuroinflammation after SAH by activating BMVECs pyroptosis triggered by caspase-1. Therefore, RIG-I can serve as a potential therapeutic target for EBI after SAH.

## Ethical approval

Animal studies were approved by the ethics committee of First Affliated Hospital of Harbin Medical University. This study was performed in strict accordance with the NIH guidelines for the care and use of laboratory animals (NIH Publication No. 85-23 Rev. 1985).

## Author contributions

Bowen Sun, MD, PhD: Drafting of the manuscript, major role in the acquisition of data, conception and design of study. Yuchen Li, MD, PhD: Drafting of the manuscript, major role in the acquisition of data, analysis of study design. Shuai Lan, MD: Contributed to the and ​*in vivo* ​experiments of the study. Xi-ao Wang, MD: Contributed to the molecular biology experiments of the study. Yeping Ling, MD: Contributed to the analyzation of the data. Harshal Sawant, PhD: Contributed to the ​*in vitro* ​experiments of the study. Bohan Zhang, MD, PhD: Contributed to the analysis of the research and molecular biology experiments of the study. Jinshuo Yang, MD: Contributed to the analysis of the research and animal behavior test of the study. Jinju Wang, PhD: Contributed to the analysis of the research. Pei Wu, MD, PhD: Contributed to the supervision of the study process. Shancai Xu, MD, PhD: Drafting of the manuscript, major role in the acquisition of data, analysis of study. Ji Bihl, MD, PhD: Drafting of the manuscript, major role in the acquisition of data, conception design of study and supervising the process of the study. Huaizhang Shi, MD, PhD: Drafting of the manuscript, major role in the acquisition of data, analysis of study design.

## Funding

This research was funded by the Postdoctoral Fund Project of Heilongjiang Province (LBH-Z20001), Project of China Postdoctoral Science Foundation Project (2021MD703829), Youth Project of National Natural Science Foundation of China (82101383), the Outstanding Youth Project of Natural Science Foundation of Heilongjiang Province (YQ2023H008), the Young Medical Talent Funding Project of The First Affiliated Hospital of Harbin Medical University (No. 2021Y10), the Wu Jieping Medical Foundation (320.6750.2021-04-61), the Heilongjiang Province Key R&D Plan Project (2022ZX06C03), the Research Innovation Fund of the First Affiliated Hospital of Harbin Medical University (2023M04), the Pilot grants from WV-CTSI (National Institute of General Medical Sciences, U54GM104942) and WV-INBRE (P20GM103434).

## Declaration of competing interest

The authors declare that they have no known competing financial interests or personal relationships that could have appeared to influence the work reported in this paper.

## References

[1] Claassen J., Park S. (2022). Spontaneous subarachnoid haemorrhage. Lancet.

[2] Macdonald R.L., Schweizer T.A. (2017). Spontaneous subarachnoid haemorrhage. Lancet.

[3] Martin S.S., Aday A.W., Almarzooq Z.I., Anderson C.A.M., Arora P., Avery C.L. (2024). 2024 heart disease and stroke statistics: a report of us and global data from the American heart association. Circulation.

[4] Hoh B.L., Ko N.U., Amin-Hanjani S., Chou S.-Y., Cruz-Flores S., Dangayach N.S. (2023). 2023 guideline for the management of patients with aneurysmal subarachnoid hemorrhage: a guideline from the American heart association/American stroke association. Stroke.

[5] Lauzier D.C., Jayaraman K., Yuan J.Y., Diwan D., Vellimana A.K., Osbun J.W. (2023). Early brain injury after subarachnoid hemorrhage: incidence and mechanisms. Stroke.

[6] de Winkel J., Roozenbeek B., Dijkland S.A., Dammers R., van Doormaal P.J., van der Jagt M. (2024). Personalized decision-making for aneurysm treatment of aneurysmal subarachnoid hemorrhage: development and validation of a clinical prediction tool. BMC Neurol.

[7] Wei C., Jiang W., Wang R., Zhong H., He H., Gao X. (2024). Brain endothelial GSDMD activation mediates inflammatory BBB breakdown. Nature.

[8] Zhao Z., Nelson A.R., Betsholtz C., Zlokovic B.V. (2015). Establishment and dysfunction of the blood-brain barrier. Cell.

[9] Amersfoort J., Eelen G., Carmeliet P. (2022). Immunomodulation by endothelial cells - partnering up with the immune system?. Nat Rev Immunol.

[10] Xu S., Jin T., Weng J. (2022). Endothelial cells as a key cell type for innate immunity: a focused review on RIG-I signaling pathway. Front Immunol.

[11] Chen Y., Galea I., Macdonald R.L., Wong G.K.C., Zhang J.H. (2022). Rethinking the initial changes in subarachnoid haemorrhage: focusing on real-time metabolism during early brain injury. EBioMedicine.

[12] Liu C., Yao K., Tian Q., Guo Y., Wang G., He P. (2023). CXCR4-BTK axis mediate pyroptosis and lipid peroxidation in early brain injury after subarachnoid hemorrhage via NLRP3 inflammasome and NF-κB pathway. Redox Biol.

[13] Xu P., Hong Y., Xie Y., Yuan K., Li J., Sun R. (2021). TREM-1 exacerbates neuroinflammatory injury via NLRP3 inflammasome-mediated pyroptosis in experimental subarachnoid hemorrhage. Transl Stroke Res.

[14] Hu Q., Zhang R., Dong X., Yang D., Yu W., Du Q. (2024). Huperzine A ameliorates neurological deficits after spontaneous subarachnoid hemorrhage through endothelial cell pyroptosis inhibition. Acta Biochim Biophys Sin.

[15] Baris A., Fraile-Bethencourt E., Eubanks J., Khou S., Anand S. (2023). Thymidine phosphorylase facilitates retinoic acid inducible gene-I induced endothelial dysfunction. Cell Death Dis.

[16] Wang P.T., Li N., Wang X.Y., Chen J.L., Geng C.H., Liu Z.Q. (2021). RIG-I, a novel DAMPs sensor for myoglobin activates NF-κB/caspase-3 signaling in CS-AKI model. Mil Med Res.

[17] de Rivero Vaccari J.P., Brand F.J., Sedaghat C., Mash D.C., Dietrich W.D., Keane R.W. (2014). RIG-1 receptor expression in the pathology of Alzheimer's disease. J Neuroinflammation.

[18] Zhou H.J., Li H., Shi M.Q., Mao X.N., Liu D.L., Chang Y.R. (2017). Protective effect of Klotho against ischemic brain injury is associated with inhibition of RIG-I/NF-κB signaling. Front Pharmacol.

[19] Walsh J.G., Muruve D.A., Power C. (2014). Inflammasomes in the CNS. Nat Rev Neurosci.

[20] Israelov H., Ravid O., Atrakchi D., Rand D., Elhaik S., Bresler Y. (2020). Caspase-1 has a critical role in blood-brain barrier injury and its inhibition contributes to multifaceted repair. J Neuroinflammation.

[21] Fang Y., Wang X., Lu J., Shi H., Huang L., Shao A. (2022). Inhibition of caspase-1-mediated inflammasome activation reduced blood coagulation in cerebrospinal fluid after subarachnoid haemorrhage. EBioMedicine.

[22] Poeck H., Bscheider M., Gross O., Finger K., Roth S., Rebsamen M. (2010). Recognition of RNA virus by RIG-I results in activation of CARD9 and inflammasome signaling for interleukin 1 beta production. Nat Immunol.

[23] Li N., Chen J., Geng C., Wang X., Wang Y., Sun N. (2022). Myoglobin promotes macrophage polarization to M1 type and pyroptosis via the RIG-I/Caspase1/GSDMD signaling pathway in CS-AKI. Cell Death Dis.

[24] van Swieten J.C., Koudstaal P.J., Visser M.C., Schouten H.J., van Gijn J. (1988). Interobserver agreement for the assessment of handicap in stroke patients. Stroke.

[25] Percie du Sert N., Hurst V., Ahluwalia A., Alam S., Avey M.T., Baker M. (2020). The ARRIVE guidelines 2.0: updated guidelines for reporting animal research. PLoS Biol.

[26] Ma S.-J., Li C., Yan C., Liu N., Jiang G.-Y., Yang H.-R. (2023). Melatonin alleviates early brain injury by inhibiting the NRF2-mediated ferroptosis pathway after subarachnoid hemorrhage. Free Radic Biol Med.

[27] Liu B., Tian Y., Li Y., Wu P., Zhang Y., Zheng J. (2022). ACEA attenuates oxidative stress by promoting mitophagy via CB1R/Nrf1/PINK1 pathway after subarachnoid hemorrhage in rats. Oxid Med Cell Longev.

[28] Xu P., Tao C., Zhu Y., Wang G., Kong L., Li W. (2021). TAK1 mediates neuronal pyroptosis in early brain injury after subarachnoid hemorrhage. J Neuroinflammation.

[29] Tian Y., Liu B., Li Y., Zhang Y., Shao J., Wu P. (2022). Activation of RARα receptor attenuates neuroinflammation after SAH via promoting M1-to-M2 phenotypic polarization of microglia and regulating Mafb/Msr1/PI3K-Akt/NF-κB pathway. Front Immunol.

[30] Cao S., Shrestha S., Li J., Yu X., Chen J., Yan F. (2017). Melatonin-mediated mitophagy protects against early brain injury after subarachnoid hemorrhage through inhibition of NLRP3 inflammasome activation. Sci Rep.

[31] Lu J., Sun Z., Fang Y., Zheng J., Xu S., Xu W. (2019). Melatonin suppresses microglial necroptosis by regulating deubiquitinating enzyme A20 after intracerebral hemorrhage. Front Immunol.

[32] Wang Y., Yang X., Cao Y., Li X., Xu R., Yan J. (2023). Electroacupuncture promotes remyelination and alleviates cognitive deficit via promoting OPC differentiation in a rat model of subarachnoid hemorrhage. Metab Brain Dis.

[33] Cheng M., Liu L., Zhang T., Chen Y., Wang Q., Wu Y. (2022). Extracellular vesicles derived from bone marrow mesenchymal stem cells alleviate neurological deficit and endothelial cell dysfunction after subarachnoid hemorrhage via the KLF3-AS1/miR-83-5p/TCF7L2 axis. Exp Neurol.

[34] He T., Xiong J., Huang Y., Zheng C., Liu Y., Bi X. (2019). Klotho restrain RIG-1/NF-κB signaling activation and monocyte inflammatory factor release under uremic condition. Life Sci.

[35] Chiang C., Beljanski V., Yin K., Olagnier D., Ben Yebdri F., Steel C. (2015). Sequence-specific modifications enhance the broad-spectrum antiviral response activated by RIG-I agonists. J Virol.

[36] Kumpunya S., Thim-Uam A., Thumarat C., Leelahavanichkul A., Kalpongnukul N., Chantaravisoot N. (2022). cGAS deficiency enhances inflammasome activation in macrophages and inflammatory pathology in pristane-induced lupus. Front Immunol.

[37] Liu W.-B., Wang S.-S., Zhang X., Ke Z.-Z., Wen X.-Y., Zhao J. (2024). Enhanced cardiomyocyte NLRP3 inflammasome-mediated pyroptosis promotes d-galactose-induced cardiac aging. J Am Heart Assoc.

[38] Li Y., Wang J., Chen S., Wu P., Xu S., Wang C. (2020). miR-137 boosts the neuroprotective effect of endothelial progenitor cell-derived exosomes in oxyhemoglobin-treated SH-SY5Y cells partially via COX2/PGE2 pathway. Stem Cell Res Ther.

[39] Chen R., Wen D., Fu W., Xing L., Ma L., Liu Y. (2022). Treatment effect of DNA framework nucleic acids on diffuse microvascular endothelial cell injury after subarachnoid hemorrhage. Cell Prolif.

[40] Lin J., Xu Y., Guo P., Chen Y.-J., Zhou J., Xia M. (2023). CCL5/CCR5-mediated peripheral inflammation exacerbates blood‒brain barrier disruption after intracerebral hemorrhage in mice. J Transl Med.

[41] Wu Q., Qi L., Li H., Mao L., Yang M., Xie R. (2017). Roflumilast reduces cerebral inflammation in a rat model of experimental subarachnoid hemorrhage. Inflammation.

[42] Oladapo A., Jackson T., Menolascino J., Periyasamy P. (2024). Role of pyroptosis in the pathogenesis of various neurological diseases. Brain Behav Immun.

[43] Zhen H., Hu Y., Liu X., Fan G., Zhao S. (2024). The protease caspase-1: activation pathways and functions. Biochem Biophys Res Commun.

[44] Hirsch Y., Geraghty J.R., Katz E.A., Testai F.D. (2021). Inflammasome caspase-1 activity is elevated in cerebrospinal fluid after aneurysmal subarachnoid hemorrhage and predicts functional outcome. Neurocritical Care.

[45] Peeyush Kumar T., McBride D.W., Dash P.K., Matsumura K., Rubi A., Blackburn S.L. (2019). Endothelial cell dysfunction and injury in subarachnoid hemorrhage. Mol Neurobiol.

[46] Liu C., Yao K., Tian Q., Guo Y., Wang G., He P. (2023). CXCR4-BTK axis mediate pyroptosis and lipid peroxidation in early brain injury after subarachnoid hemorrhage via NLRP3 inflammasome and NF-κB pathway. Redox Biol.

[47] Yoneyama M., Kato H., Fujita T. (2024). Physiological functions of RIG-I-like receptors. Immunity.

[48] Jiang Y., Zhang H., Wang J., Chen J., Guo Z., Liu Y. (2023). Exploiting RIG-I-like receptor pathway for cancer immunotherapy. J Hematol Oncol.

[49] Zhang C., Zhang S., Wang L., Kang S., Ma J., Liu S. (2021). The RIG-I signal pathway mediated Panax notoginseng saponin anti-inflammatory effect in ischemia stroke. Evid Based Complement Alternat Med.

[50] Zhou H.-J., Li H., Shi M.-Q., Mao X.-N., Liu D.-L., Chang Y.-R. (2017). Protective effect of Klotho against ischemic brain injury is associated with inhibition of RIG-I/NF-κB signaling. Front Pharmacol.

[51] Asdonk T., Motz I., Werner N., Coch C., Barchet W., Hartmann G. (2012). Endothelial RIG-I activation impairs endothelial function. Biochem Biophys Res Commun.

[52] Ma B., Dela Cruz C.S., Hartl D., Kang M.-J., Takyar S., Homer R.J. (2011). RIG-like helicase innate immunity inhibits vascular endothelial growth factor tissue responses via a type I IFN-dependent mechanism. Am J Respir Crit Care Med.

[53] Liu F., Wu S., Ren H., Gu J. (2011). Klotho suppresses RIG-I-mediated senescence-associated inflammation. Nat Cell Biol.

[54] Chen S., Guo D., Zhu Y., Xiao S., Xie J., Zhang Z. (2024). Amyloid β oligomer induces cerebral vasculopathy via pericyte-mediated endothelial dysfunction. Alzheimers Res Ther.

[55] Nirwane A., Kang M., Adithan A., Maharaj V., Nguyen F., Santaella Aguilar E. (2024). Endothelial and mural laminin-α5 contributes to neurovascular integrity maintenance. Fluids Barriers CNS.

[56] Gu L., Chen H., Geng R., Liang T., Chen Y., Wang Z. (2024). Endothelial pyroptosis-driven microglial activation in choroid plexus mediates neuronal apoptosis in hemorrhagic stroke rats. Neurobiol Dis.

