# Supplementary material for: Retinoic acid-inducible gene-I aggravates neuroinflammation in early brain injury after subarachnoid hemorrhage through mediating brain microvascular endothelial cell pyroptosis
Source: Neurotherapeutics. 2025 Apr 2;22(4):e00572. doi: 10.1016/j.neurot.2025.e00572 (PMC12418424; doi:10.1016/j.neurot.2025.e00572)
Supplement: Multimedia component 1 [file mmc1.zip › Supplement/Additional figure 1.docx]

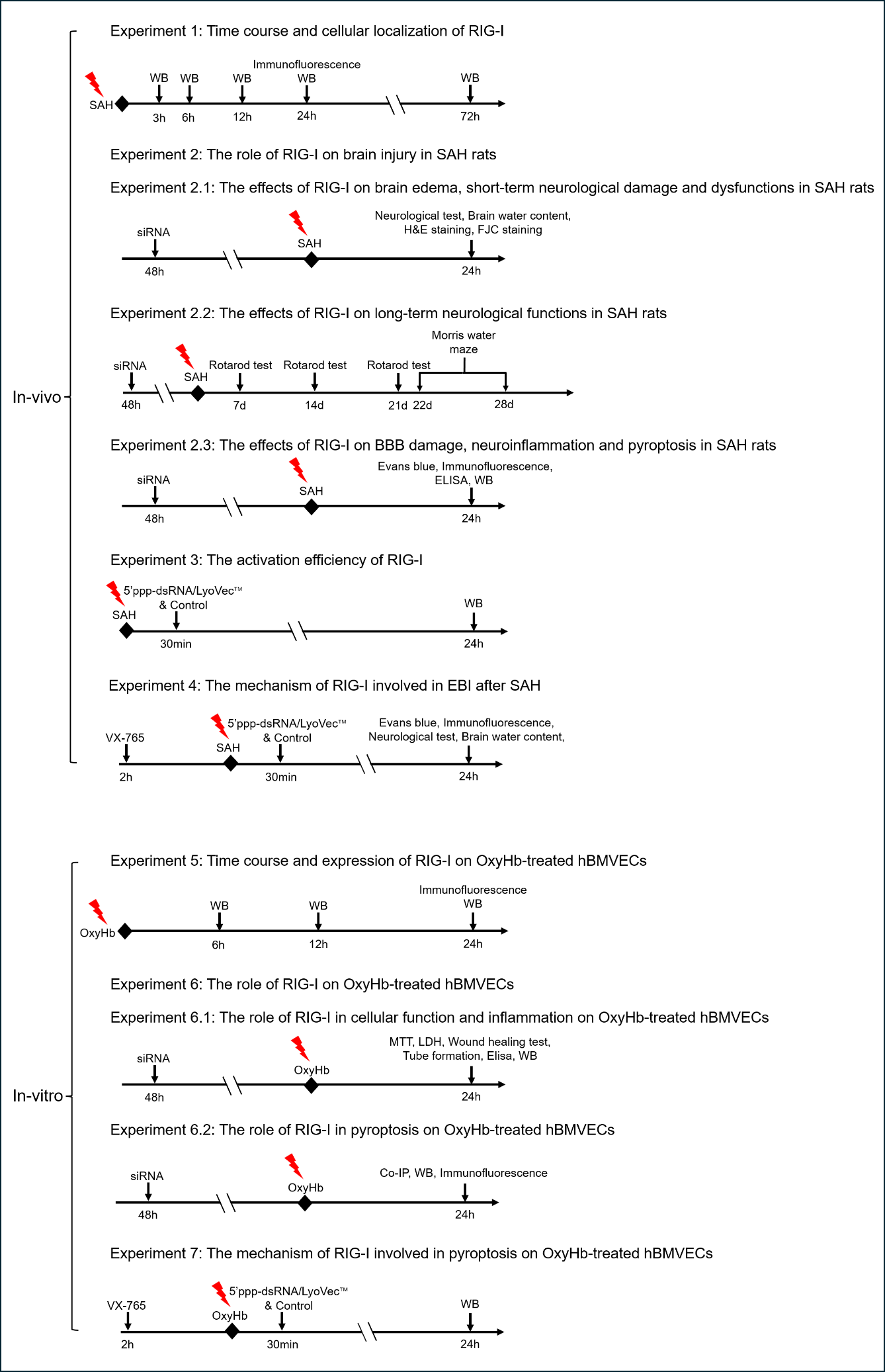


Figure S1. Experiment design. WB,Western Blot；H&E staining,Hematoxylin-Eosin Staining；FJC staining,Fluoro-Jade C Staining；ELISA,Enzyme-Linked Immunosorbent Assay；OxyHb，Oxyhemaglobin；MTT Assay,3- (4,5-Dimethylthiazol-2-yl)-2,5-Diphenyltetrazolium Bromide Assay；LDH,Lactate Dehydrogenase Test；Co-IP,Co-immunoprecipitation.

**Experiment 1**

To assess the expression levels and localization of RIG-I in endothelial cells after SAH, animals were randomly divided into six groups: sham (n=9), where rats underwent surgery without internal carotid artery puncture; 6h post-SAH (n=6); 12 post-SAH (n=6); 24 post-SAH (n=9); and 72 post-SAH (n=6). At the predetermined time points, the brain tissues from the injured hemisphere temporal lobes were collected for Western blotting (WB) to evaluate the level of RIG-I expression (n=6). Additionally, three rats were taken from each of the Sham group and the 24-hour post-SAH group to determine RIG-I expression in endothelial cells of the injured side temporal lobe through Double immunofluorescence labeling method (Fig. S2).

**Experiment 2**

To assess the role of RIG-I in brain injury following SAH, rats were randomly divided into four groups: (1) sham; (2) SAH; (3) SAH + siRNA-Negative Control (NC), where rats received intracerebroventricular injection of control siRNA 48 hours before SAH modeling; (4) SAH + RIG-I siRNA group (n=12), where rats received intracerebroventricular injection of 500 pmol RIG-I siRNA 48 hours before SAH modeling

**2.1**

To evaluate the role of RIG-I in cerebral edema, histological damage, and short-term neurological function after SAH, rats were randomly divided into 4 groups: (1) Sham (n=12); (2) SAH (n=12); (3) SAH + si-NC (n=12); (4) SAH + si-RIG-I (n=12), with the same modeling methods as described above. 6 rats from each group were selected for neurological function assessment (beam balance & modified Garcia score) 24h after SAH. Sacrifice and brain water weight measurement are processed after the neurological function assessment. Additionally, 3 rats from each group were randomly selected for H&E staining, and 3 rats were selected for FJC staining.

**2.2**

To evaluate the role of RIG-I in long-term neurological function after SAH, rats were randomly divided into four groups: (1) Sham (n=10); (2) SAH (n=10); (3) SAH + si-NC (n=10); (4) SAH + si-RIG-I (n=10), with same modeling methods as mentioned above. Each group of 10 rats underwent Rotarod testing on days 7, 14, and 21 post-SAH.Morris water maze test was conducted on the 22nd to 28th day post SAH.

**2.3**

To evaluate the role of RIG-I in blood-brain barrier disruption, neuroinflammation, and endothelial cell pyroptosis following subarachnoid hemorrhage (SAH), rats were randomly divided into four groups: (1) Sham surgery group (n=21); (2) SAH group (n=21); (3) SAH + si-NC group (n=21); (4) SAH + si-RIG-I group (n=21). The modeling methods for each group were the same as mentioned above. Rats will be sacrificed 24 hours after SAH to collect the brains. 6 rats from each group were selected for WB to evaluate the expression level of ZO-1, MMP-9, Occludin, IL-1β, IL-6, NF-κB, p-NF-κB, Caspase-1, C-Caspase1, and GSDMD-N. 6 rats each were selected for Evans Blue extravasation test and ELISA to evaluate the level of IL-1β and TNF-α in the injured hemisphere brain tissue lysates, 3 for double immunofluorescence labeling method to evaluate neutrophil infiltration in the brain and the expression level of ZO-1 and C-Caspase-1 in endothelial cells (CD31), 3 for immunofluorescence of Evans Blue.

**Experiment 3**

To activate RIG-I, its activator 5'ppp-dsRNA was injected into the ventricle 30 minutes after SAH modeling. Randomly divide 30 rats into the Sham group; SAH+5'ppp-dsRNA control group, SAH+5'ppp-dsRNA (5μg/kg) group, SAH+5'ppp-dsRNA (10μg/kg) group, and SAH+5'ppp-dsRNA (20μg/kg) group. Subsequently, brain tissue proteins were collected for Western blot analysis of the activation level of RIG-I.

**Experiment 4**

To further clarify the mechanism, in addition to injecting 5'ppp dsRNA after modeling, Caspase-1 inhibitor VX-765 was injected into the ventricle 2 hours before modeling. Randomly divide 90 rats into three groups: Sham group, SAH+5'ppp-dsRNA control group, SAH+5'ppp-dsRNA (5μg/kg) group, SAH+5'ppp-dsRNA(5μg/kg) +vehicle group, and SAH+5'ppp-dsRNA(5μg/kg) +VX-765 group. After 24 hours of modeling, 6 rats were selected from each group for neurological function evaluation (beam balance and modified Garcia score). After neurological function evaluation, the rats were euthanized for brain water weight measurement. Six rats were randomly selected from each group to evaluate BBB permeability using Evans Blue extravasation, and another six rats were subjected to ELISA assay to detect the levels of apoptosis markers and inflammatory factors in brain tissue lysate. In addition, routine assessment of bleeding volume is required after euthanizing rats.

**Experiment 5**

To further verify the role of RIG-I in brain microvascular endothelial cells, an in vitro SAH model was established by stimulating hBMVEC cell line with 10 μM oxyhemoglobin (OxyHb). To evaluate the expression of RIG-I after OxyHb treatment, cells were randomly divided into 4 groups: Control, 6h OxyHb, 12h OxyHb, and 24h OxyHb. Cell proteins were collected at the corresponding time points for WB analysis to evaluate RIG-I levels. Immunofluorescence staining method was also used to evaluate RIG-I expression. Each experiment was repeated for six times.

**Experiment 6**

To verify the role of RIG-I in hBMVECs cell line treated with OxyHb, cells were randomly divided into four groups: Control, OxyHb, OxyHb+siNC, and Oxy+siRIG-I. siNC or siRIG-I was added 24 hours before OxyHb treatment to knock down the expression of RIG-I, and experiments were conducted 24 hours after OxyHb treatment.

**Experiment 6.1**

To determine the role RIG-I plays in cell viability, function, and inflammation in OxyHb treated hBMVECs cell line, we conducted the following experiments. MTT assay is used to evaluate cell viability, while LDH assay is used to assess the cytotoxicity. Tube formation assay and Wound Healing test are conducted to evaluate cell function. Cell protein and culture medium supernatant are respectively used for WB and ELISA to determine the levels of classic inflammation markers such as IL-1β, IL-6, NF-κB, and p-NF-κB.

**Experiment 6.2**

To determine the role of RIG-I in pyroptosis in OxyHb-treated hBMVECs cell line and its interaction with Caspase-1, we conducted the following experiments. Co-IP is used to verify the interaction between RIG-I and Caspase-1, while WB and immunofluorescence staining are employed to determine the levels of pyroptosis proteins such as Caspase-1, C-Caspase1, and GSDMD-N.

**Experiment 7**

To verify the role RIG-I plays in pyroptosis in OxyHb treated hBMVECs cell line, Caspase-1 inhibitor VX-765 was applied 2 hours before the treatment of OxyHb to inhibit the expression of Caspase1. RIG-I activator 5’ppp-dsRNA/LyoVecTM was applied 30 minutes after the treatment of OxyHb to activate the expression of RIG-I. Cells were randomly divided into the following groups: Control, OxyHb+5’ppp-dsRNA control, OxyHb+5’ppp-dsRNA, Oxy+5’ppp-dsRNA+Vehicle, and Oxy+5’ppp-dsRNA+VX-765.
