# Supplementary material for: Retinoic acid-inducible gene-I aggravates neuroinflammation in early brain injury after subarachnoid hemorrhage through mediating brain microvascular endothelial cell pyroptosis
Source: Neurotherapeutics. 2025 Apr 2;22(4):e00572. doi: 10.1016/j.neurot.2025.e00572 (PMC12418424; doi:10.1016/j.neurot.2025.e00572)
Supplement: Multimedia component 1 [file mmc1.zip › Supplement/Additional figure 6.docx]

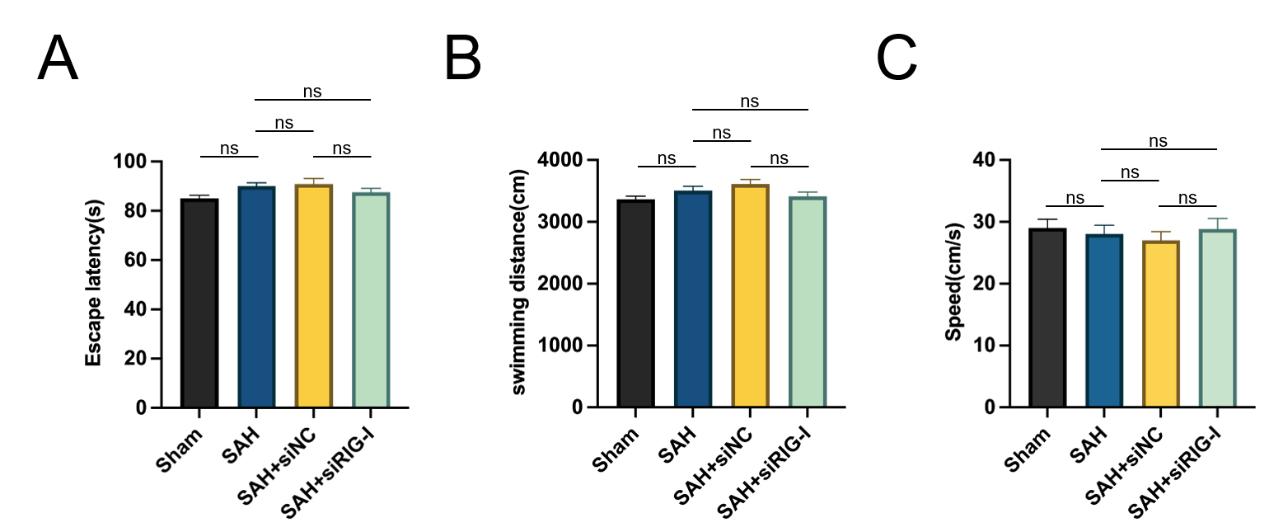


Supplementary Figure S6. Supplementary content for water maze test. (A) Escape latency in the visible platform trial of the water maze test. n=6 per group. (B-C) Swimming distance and speed in the probe trial of the water maze test. n=6 per group. ns: not significant.
