# Supplementary material for: Retinoic acid-inducible gene-I aggravates neuroinflammation in early brain injury after subarachnoid hemorrhage through mediating brain microvascular endothelial cell pyroptosis
Source: Neurotherapeutics. 2025 Apr 2;22(4):e00572. doi: 10.1016/j.neurot.2025.e00572 (PMC12418424; doi:10.1016/j.neurot.2025.e00572)
Supplement: Multimedia component 1 [file mmc1.zip › Supplement/Additional figure 9.docx]

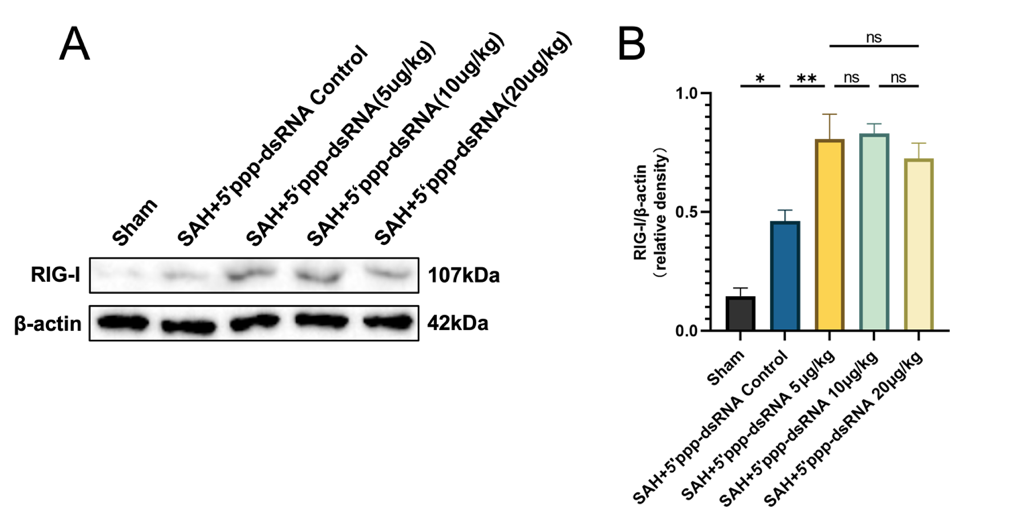


Supplementary Figure S9. (A-B) Representative western blot images and densitometric quantification of the expression of RIG-I. *: P<0.05, **: P<0.01. ns, not significant.
