# Supplementary material for: Retinoic acid-inducible gene-I aggravates neuroinflammation in early brain injury after subarachnoid hemorrhage through mediating brain microvascular endothelial cell pyroptosis
Source: Neurotherapeutics. 2025 Apr 2;22(4):e00572. doi: 10.1016/j.neurot.2025.e00572 (PMC12418424; doi:10.1016/j.neurot.2025.e00572)
Supplement: Multimedia component 1 [file mmc1.zip › Supplement/Additional tables.docx]

**Supplementary Table S1 to S4**

Supplementary Table S1. mRS score

Supplementary Table S2. Modified Garcia score.

Supplementary Table S3. Beam balance test.

Supplementary Table S4. Antibodies used in this study.

Supplementary Table S5. Distribution of animals according to different groups and mortality rate.

**Supplementary Table 1: The Modified Rankin Scale**

| Grade | Description |
| --- | --- |
| 0 | No symptoms at all |
| 1 | No significant disability despite symptoms: able to carry out all usual duties and activities |
| 2 | Slight disability: unable to carry out all previous activities but able to look after own affairs without assistance |
| 3 | Moderate disability: requiring some help, but able to walk without assistance |
| 4 | Moderately severe disability: unable to walk without assistance, and unable to attend to own bodily needs without assistance |
| 5 | Severe disability: bedridden, incontinent, and requiring constant nursing care and attention |

**Supplementary Table S2: Modified Garcia score.**

| **Test** | **Score** | | | |
| --- | --- | --- | --- | --- |
|  | **0** | **1** | **2** | **3** |
| Spontaneous Activity (in a cage for 5 min) | No movement | Barely moves position | Moves but does not approach at least three sides of the cage | Moves and approaches at least three sides of the cage |
| Spontaneous movements of all limbs | No movement | Slight movement of limbs | Moves all limbs but slowly | Move all limbs same as pre-SAH |
| Movements of forelimbs (outstretching while held by the tail) | No movement | Slight outreaching | Outreach is limited and less than pre-SAH | Outreach same as pre-SAH |
| Climbing the wall of the wire cage | - | Fails to climb | Climbs weakly | Normal climbing |
| Reaction to touch on both sides of the trunk | - | No response | Weak response | Normal response |
| Response to vibrissae touch | - | No response | Weak response | Normal response |

**Supplementary Table S3: Beam balance test.**

| **Score** | **Description: Beam walking (60 sec)** |
| --- | --- |
| 0 | No walking and falls off |
| 1 | No walking but remains on beam |
| 2 | Walking but falls off |
| 3 | Walking less than 20 cm |
| 4 | Walking beyond 20 cm |

**Supplementary Table S4: Antibodies used in this study.**

| **Antibody** | **Manufacturer** | **Catalog number** | **Dilution** |
| --- | --- | --- | --- |
| anti-RIG-I | Santa Cruz | sc-376845 | 1:100 |
| anti-GSDMD-N | Abcam | ab215203  ab219800 | 1:1000  1:1000 |
| anti-ZO-1 | Santa Cruz | sc-33725 | 1:200 |
| anti-Occludin | Santa Cruz | sc-133256 | 1:200 |
| anti-MMP9 | Santa Cruz | sc-13520 | 1:200 |
| anti-NF-kB-p65 | Cell Signaling Tech | 8242 | 1:1000 |
| anti-p-NF-kB p65 | Cell Signaling Tech | 3033 | 1:1000 |
| anti- caspase-1 | Invitrogen | MA5-16215 | 1:500 |
| anti-cleaved caspase-1 | Invitrogen | PA5-77886 | 1:500 |
| anti-IL-6 | Invitrogen | PA5-144595 | 1:500 |
| anti-IL-1β | Invitrogen | PA5-88078 | 1:500 |
| anti-β-actin | Abcam | ab8226 | 1:2000 |
| Goat anti-Mouse IgG (H+L) Secondary Antibody, HRP | Invitrogen | 31430 | 1:5000 |
| Goat anti-Rabbit IgG (H+L) Secondary Antibody, HRP | Invitrogen | 31460 | 1:5000 |
| Donkey Anti-Goat IgG H&L (FITC) preadsorbed ab7121 | Abcam | ab7127 | 1:200 |
| Dylight594-AffiniPure Donkey Anti-Mouse IgG (H+L) | BOSTER Biological Technology co. ltd | BA1148 | 1:200 |
| TRITC Conjugated AffiniPure Goat Anti-rabbit IgG (H+L) | BOSTER Biological Technology co. ltd | BA1090 | 1:200 |
| **Antibody** | **Manufacturer** | **Catalog number** | |
| Human TNF alpha ELISA Kit | Abcam | ab181421 | |
| Rat TNF alpha ELISA Kit | Abcam | ab236712 | |
| Human IL-1 beta ELISA Kit | Abcam | ab214025 | |
| Rat IL-6 ELISA Kit | Abcam | ab234570 | |
| Human Caspase-1 ELISA Kit | Invitrogen | EH70RB | |
| Human RIG-I ELISA Kit | Camilo | 2H-KMLJh311924 | |

**Table S5. Distribution of animals according to different groups and mortality rate**

|  | **Neurological test** | |  |  |  |  |  |  |  |  |  |  |
| --- | --- | --- | --- | --- | --- | --- | --- | --- | --- | --- | --- | --- |
|  | **Short-term** | **Long-term** | **Brain water content** | **Evans blue** | **WB** | **IF** | **H&E** | **ELISA** | **Death** | **Excluded** | **Total** | **Mortality** |
| **Experiment 1** | | | | | | | | | | | | |
| Sham |  |  |  |  | 6 | 3 |  |  | 0 | 0 | 9 | 0.00% |
| SAH 3h |  |  |  |  | 6 |  |  |  | 1 | 1 | 7 | 14.29% |
| SAH 6h |  |  |  |  | 6 |  |  |  | 1 | 0 | 7 | 14.29% |
| SAH 12h |  |  |  |  | 6 |  |  |  | 1 | 1 | 7 | 14.29% |
| SAH 24h |  |  |  |  | 6 | 3 |  |  | 2 | 1 | 11 | 18.18% |
| SAH 72h |  |  |  |  | 6 |  |  |  | 1 | 0 | 7 | 14.29% |
| **Experiment 2.1** | | | | | | | | | | | | |
| Sham | 6 |  | 6^*^ |  |  | 3^#^ | 3 |  | 0 | 0 | 9^$^ | 0.00% |
| SAH | 6 |  | 6^*^ |  |  | 3^#^ | 3 |  | 3^$^ | 0 | 12^$^ | 25.00% |
| SAH+siNC | 6 |  | 6^*^ |  |  | 3 | 3 |  | 4^$^ | 1 | 16^$^ | 25.29% |
| SAH+siRIG-I | 6 |  | 6^*^ |  |  | 3 | 3 |  | 2^$^ | 1 | 14^$^ | 14.29% |
| **Experiment 2.2** | | | | | | | | | | | | |
| Sham |  | 10 |  |  |  |  |  |  | 0 | 0 | 10 | 0.00% |
| SAH |  | 10 |  |  |  |  |  |  | 3 | 1 | 13 | 23.08% |
| SAH+siNC |  | 10 |  |  |  |  |  |  | 3 | 2 | 13 | 23.08% |
| SAH+siRIG-I |  | 10 |  |  |  |  |  |  | 3 | 0 | 13 | 23.08% |
| **Experiment 2.3** | | | | | | | | | | | | |
| Sham |  |  |  | 9 | 6 | 3^#^ |  | 6 | 0 | 0 | 21^$^ | 0.00% |
| SAH |  |  |  | 9 | 6 | 3^#^ |  | 6 | 3^$^ | 3 | 24^$^ | 14.29% |
| SAH+siNC |  |  |  | 9 | 6 | 3 |  | 6 | 6 | 3 | 30 | 22.22% |
| SAH+siRIG-I |  |  |  | 9 | 6 | 3 |  | 6 | 4 | 3 | 28 | 16.00% |
| **Experiment 3** | | | | | | | | | | | | |
| Sham |  |  |  |  | 6 |  |  |  | 0 | 0 | 6 | 0.00% |
| SAH+5’ppp-dsRNA-control |  |  |  |  | 6 |  |  |  | 1 | 1 | 7 | 14.29% |
| SAH+5’ppp-dsRNA(5μg/kg) |  |  |  |  | 6 |  |  |  | 2 | 1 | 8 | 25.00% |
| SAH+5’ppp-dsRNA(10μg/kg) |  |  |  |  | 6 |  |  |  | 1 | 0 | 7 | 14.29% |
| SAH+5’ppp-dsRNA(20μg/kg) |  |  |  |  | 6 |  |  |  | 1 | 0 | 7 | 14.29% |
| **Experiment 4** | | | | | | | | | | | | |
| Sham | 6 |  | 6^*^ | 6 |  |  |  | 6 | 0 | 0 | 18 | 0.00% |
| SAH+5’ppp-dsRNA-control | 6 |  | 6^*^ | 6 |  |  |  | 6 | 5 | 1 | 23 | 21.74% |
| SAH+5’ppp-dsRNA(5μg/kg) | 6 |  | 6^*^ | 6 |  |  |  | 6 | 4 | 2 | 22 | 18.18% |
| SAH+5’ppp-dsRNA(5μg/kg) +Vehicle | 6 |  | 6^*^ | 6 |  |  |  | 6 | 3 | 2 | 21 | 14.29% |
| SAH+5’ppp-dsRNA(5μg/kg) +VX-765 | 6 |  | 6^*^ | 6 |  |  |  | 6 | 4 | 1 | 22 | 18.18% |
| **SUM** | | | | | | | | | | | | |
| Sham | 73 | | | | | | | | 0 | 0 | 73 | 0.00% |
| SAH | 261 | | | | | | | | 58 | 25 | 319 | 18.18% |
| SUM | 334 | | | | | | | | 58 | 25 | 392 | 14.80% |

*Share with short-term neurologic testing; #, Share with experiment 1; $, Rats shared with other experiments were not double counted.

Table S6. Patient information for cerebrospinal fluid analysis.

| No. | Group | Gender | Age | mRS |
| --- | --- | --- | --- | --- |
| 1 | SAH | M | 72 | 2 |
| 2 | SAH | F | 61 | 4 |
| 3 | SAH | M | 63 | 1 |
| 4 | SAH | M | 50 | 1 |
| 5 | SAH | F | 62 | 2 |
| 6 | SAH | M | 54 | 5 |
| 7 | SAH | F | 56 | 2 |
| 8 | SAH | F | 62 | 0 |
| 9 | SAH | F | 57 | 1 |
| 10 | SAH | F | 70 | 1 |
| 11 | SAH | M | 61 | 2 |
| 12 | SAH | F | 56 | 3 |
| 13 | SAH | M | 63 | 0 |
| 14 | SAH | F | 51 | 4 |
| 15 | SAH | M | 55 | 1 |
| 16 | SAH | F | 50 | 0 |
| 17 | SAH | F | 60 | 2 |
| 18 | SAH | M | 46 | 2 |
| 19 | SAH | F | 61 | 6 |
| 20 | SAH | F | 72 | 4 |
| 21 | SAH | F | 42 | 6 |
| 22 | SAH | F | 51 | 2 |
| 23 | SAH | F | 62 | 3 |
| 24 | SAH | M | 40 | 3 |
| 25 | SAH | F | 51 | 5 |
| 26 | SAH | M | 50 | 3 |
| 27 | SAH | F | 44 | 5 |
| 28 | SAH | F | 51 | 1 |
| 29 | Con | M | 36 | - |
| 30 | Con | F | 53 | - |
| 31 | Con | M | 69 | - |
| 32 | Con | M | 63 | - |
| 33 | Con | F | 54 | - |
| 34 | Con | M | 49 | - |
| 35 | Con | F | 55 | - |
| 36 | Con | F | 54 | - |
| 37 | Con | M | 58 | - |
| 38 | Con | M | 47 | - |
| 39 | Con | F | 49 | - |
| 40 | Con | F | 66 | - |

SAH: subarachnoid hemorrhage; Con: control; M: male; F: female; mRS: The Modified Rankin Scale
