# Supplementary figures and images for: Retinoic acid-inducible gene-I aggravates neuroinflammation in early brain injury after subarachnoid hemorrhage through mediating brain microvascular endothelial cell pyroptosis
Source: Neurotherapeutics. 2025 Apr 2;22(4):e00572. doi: 10.1016/j.neurot.2025.e00572 (PMC12418424; doi:10.1016/j.neurot.2025.e00572)

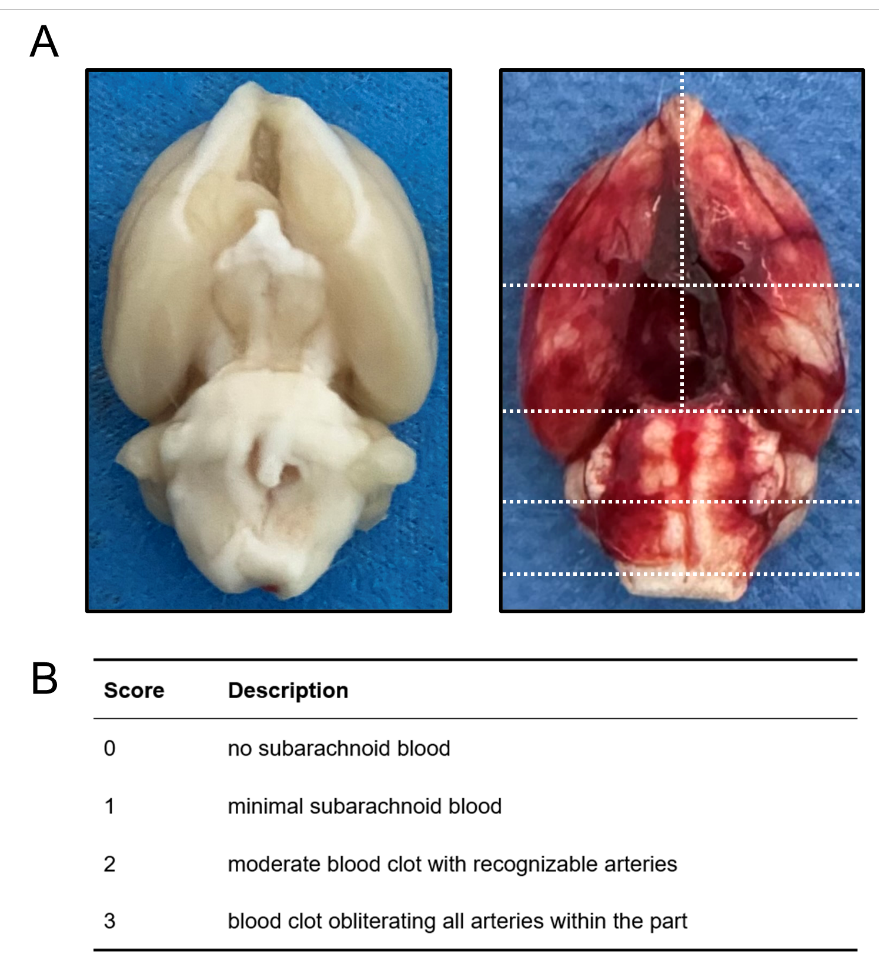


Additional Figure S2. Representative images for brain tissues and the grading system for SAH.

Supplement: Multimedia component 1 [file mmc1.zip › Supplement/Additional figure 2.docx]

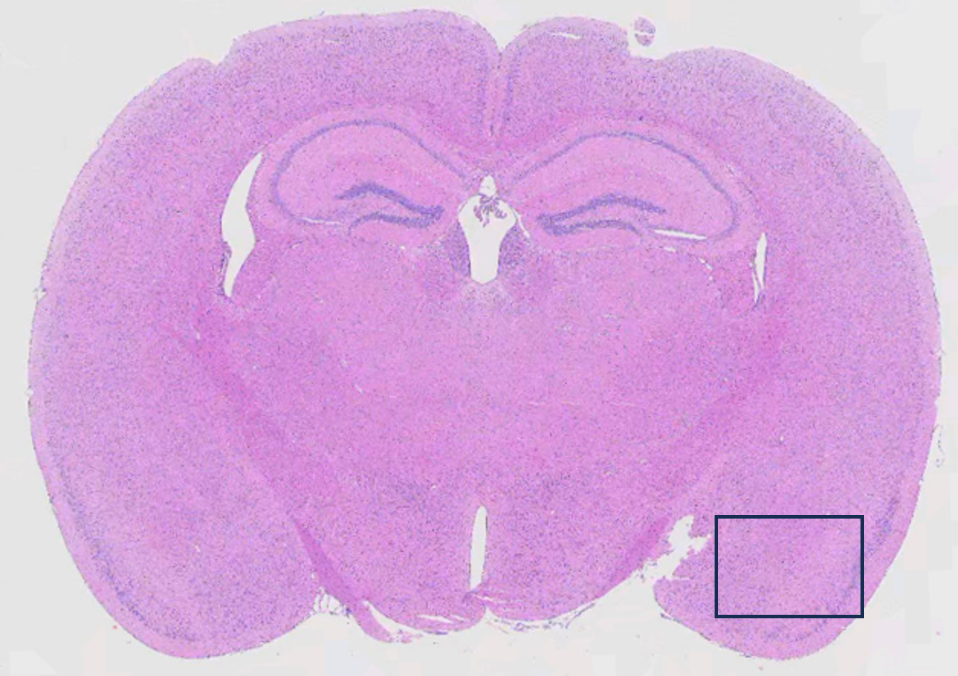


Supplementary Figure S3. Top panel indicates the location of staining (small black box).

Supplement: Multimedia component 1 [file mmc1.zip › Supplement/Additional figure 3.docx]

**
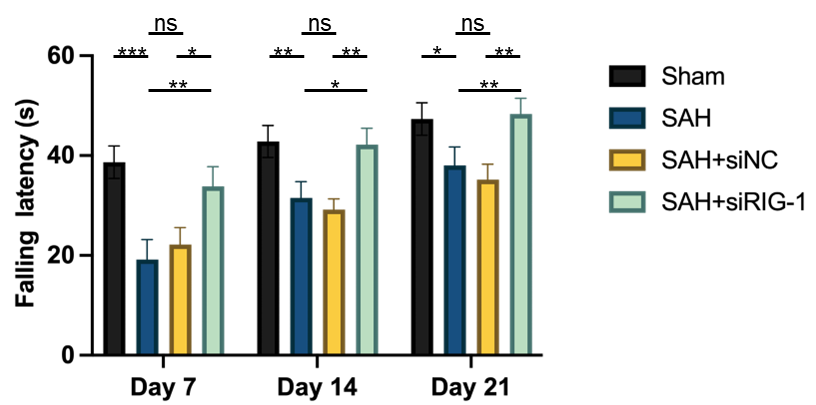
**

Supplementary Figure S5. 10-rpm rotarod test on days 7, 14, and 21 after SAH. n=10. *: P<0.05, **: P<0.01, ***: P<0.001, and ****: P<0.0001. ns, not significant.

Supplement: Multimedia component 1 [file mmc1.zip › Supplement/Additional figure 5.docx]
